# Correction: Population Biology of *Schistosoma* Mating, Aggregation, and Transmission Breakpoints: More Reliable Model Analysis for the End-Game in Communities at Risk

**DOI:** 10.1371/journal.pone.0123592

**Published:** 2015-04-01

**Authors:** The PLOS ONE Staff

## Notice of Republication

This article was republished on March 9, 2015, to correct errors in equations that were introduced during the typesetting process. Please download this article again to view the correct version. The originally published, uncorrected article and the republished, corrected article are provided here for reference.

## Supporting Information

S1 File Originally published, uncorrected article. (PDF)

S2 File Republished corrected article. (PDF)
