# Supplementary material for: Population Biology of Schistosoma Mating, Aggregation, and Transmission Breakpoints: More Reliable Model Analysis for the End-Game in Communities at Risk
Source: PLoS One. 2014 Dec 30;9(12):e115875. doi: 10.1371/journal.pone.0115875 (PMC4280120; doi:10.1371/journal.pone.0115875)
Supplement: S1 Text — S1–S5 Appendices. Extended description and formulae detailing: S1 Appendix) The background on the problems of Schistosoma transmission model formulation; S2 Appendix) Moment equations for SWB, and the extended MWB model; S3 Appendix) Mating function in Macdonald and SWB systems; S4 Appendix) Equilibria of a coupled human SWB - snail system; and S5 Appendix) Model calibration. (DOCX) [file pone.0115875.s001.docx]

# Appendix S1: Background on the problems of *Schistosoma* transmission model formulation

The classical approach to modeling *Schistosoma*-like infections exploits the mean worm burden (MWB) formulation initiated by Macdonald in 1965 and subsequently modified by others [7, 12, 17]. These equations can also be derived as a special case of the stratified (SWB) system, where host population is partitioned into burden strata $\left\{h_{n}\left(t\right)\right\}$ (hosts carrying *n* worms), if one prescribes suitable transitions rates/patterns between strata (see Figure 1). The MWB-variable in MacDonald’s formulation represents the 1^st^ moment of distribution $\left\{h_{n}\right\}$, $w\left(t\right)=\sum_{n=1}^{\infty} n h_{n}\left(t\right)$, but there are other possible extensions with higher moments [7, 30, 31, 33, 37, 38]. We review the moment equations for SWB systems in Appendix S2.

A typical “host-vector” (or “host - intermediate host”) system involves two transmission pathways and two forces of infection (FOI). For *Schistosoma*, we designate them by $\lambda$ - the snail-to-human FOI, and by $\Lambda$ - the human-to-snail FOI. To get force $\Lambda$ in a Macdonald-type MWB model, one needs to impose some *a priori* assumptions on the distribution pattern $\left\{h_{n}\right\}$, the efficiency of the worm pairing /mating process, and the resulting count of fertilized females $\left\{\psi_{n}\right\}$. The simplest assumption is $\psi_{n}=n/2$ (*i.e.,* half of adult worms = females). Then each stratum $h_{n}$ contributes $\Lambda_{n}=b\cdot n/2$, where *b* is equal to egg-production rate/female, times additional factors that account for the efficiency of human-to-snail transmission. Hence, the total snail FOI $\Lambda=b\cdot w/2$, as used in simple MacDonald-type MWB formulations.

The problem with using $\psi_{n}=n/2$ is that it can grossly overestimate$\Lambda$. If anything, such a relationship could approximately hold for heavily infected strata (*n >>* 1), provided the bulk of infection is carried by those strata. But in reality, high-burden infections are relatively few in number (small $h_{n}$ for *n>>*1), whereas the bulk of transmission is likely due to contamination by a larger pool of people having only low-intensity infections ($n=1,2,...$ ). For low *n*, worms of each sex may not be evenly distributed within human hosts, such that the likelihood of successful mating is affected by low within-host densities, even if average densities (over the larger host population) appear, at first glance, sufficient.

The need to account for uneven (aggregated) worm burden and its effects on mating behavior was recognized in early works [7, 11, 12]. Different assumptions on $\left\{h_{n}\right\}$were employed in these studies. The widely adopted distribution pattern used in later MWB studies is the negative-binomial (NB) distribution, having aggregation constant *k*, or its limiting Poisson case (when $k=\infty$). This choice of population models was based on empirical observations of human population egg count data (*e.g.,* [27-29]).

Robert May and colleagues [12, 17, 18], have conducted a systematic analysis of NB-based models with different possible (random and non-random) worm mating patterns. They derived a modified snail FOI with mating function , that depends on NB aggregation k, and the specific type of mating pattern under study (monogamous, polygamous, promiscuous, etc., see Table 2 and Figure 3). May and colleagues observed that mating function has dramatic effect on equilibria and dynamics of MWB systems. The simplest MWB system without mating () has two equilibria, a stable endemic level and unstable infection-free state, provided its basic reproductive number (BRN, defined below), $R_{0}>1$. A nontrivial mating function has two-fold effect. On the one hand, a sustained (endemic) infection requires higher overall transmission rates, hence higher BRN $R_{0}>R_{c}>1$ [40]. For an $R_{0}>R_{c}$, the system becomes bistable with two stable equilibria at “zero” and at “endemic” worm burden levels, and an intermediate unstable point or region (breakpoint), where transmission falters and below which the system naturally trends toward elimination at the “zero” point.

Overall, this suggests that obligate mating could makes transmission of infection less sustainable, hence easier to eradicate. Furthermore, the breakpoint phenomenon has important implications for model-based analysis and projections of control program outcomes [12, 33]. However, despite MacDonald’s and others’ hopeful projections that >90% reductions in snail numbers could reach a *Schistosoma* transmission breakpoint in endemic areas [7], many long term snail control projects in the 1960s were unable to interrupt transmission with less than complete snail eradication [8, 9, 16], and the existence of a practical, real breakpoint phenomenon in endemic transmission habitats remains in doubt.

The first place to look for possible discrepancies between theory and observation is the NB assumption itself, and the problem of accurately estimating the aggregation parameter *k*. It appears that *k* may vary widely for different systems and environments [28], and more importantly, it may vary for different states and transitions within the same system [35]. A proposed solution has been to treat *k* as a dynamic variable. For purposes of comparison, we have derived an extended version of the MWB system with dynamic aggregation *k* and we examined its equilibria (see Appendix S2). However, this analysis revealed some inherent inconsistencies of the NB-based methodology with either fixed or dynamic k values, particularly when applied to demographically structured populations.

One clear advantage of the SWB approach is no need for *a priori* assumptions about the distribution of infected human hosts $\left\{h_{n}\right\}$, the mean worm load, or the operative aggregation factor. In the SWB, the $\left\{h_{n}\right\}$ themselves become dynamic variables, which determine their statistics, thus the “aggregation problem” is naturally resolved. One still needs a proper estimation of mated worm counts to accurately estimate snail FOI, $\Lambda$. Our previously published version of the SWB system [23] included no mating factors, indicating that we had probably overestimated $\Lambda$ in that first analysis.

In the present paper, we revise the SWB approach to include parasite reproductive behavior, including the relevant mating ‘hurdle’, and conduct a systematic analysis of the system’s equilibria, dynamic states, and control predictions relevant to current mass-drug administration (MDA) approaches to parasite control.

# Appendix S2: Moment equations for SWB, and the extended MWB model

The basic dynamic variables for SWB are population strata $\left\{h_{n}\left(t\right):\sum_{i=0}^{\infty} h_{i}=H\left(t\right)\right\}$ (see schematic diagram Figure 1), that obey a coupled DE system [37-39]

(25)

System (25) can be recast in terms of two finite differences: forward $\Delta^{+}\left(f_{i}\right)=f_{i+1}-f_{i}$, and backward $\Delta^{-}\left(f_{i}\right)=f_{i}-f_{i-1}$,

$$\frac{dh_{i}}{dt}=-\lambda\Delta^{-}\left(h_{i}\right)+\gamma\Delta^{+}\left(ih\right)_{i}-\mu h_{i};$$

From this form, one can derive the moment equations for variables $H\left(t\right)=\sum_{i=0}^{\infty} h_{i}\left(t\right)$ - total population (0- moment); $W\left(t\right)=\sum_{i=1}^{\infty} i\cdot h_{i}\left(t\right)$ - total parasite load (1^st^ moment); $U\left(t\right)=\sum_{i=1}^{\infty} i^{2}h_{i}\left(t\right)$- 2^nd^ moment, etc. (see e.g. [33, 37])

$\begin{matrix} \frac{dH}{dt}=S_{H}-\mu H \\ \frac{dW}{dt}=S_{W}+\lambda H-\left(\gamma+\mu\right) W \\ \frac{dU}{dt}=S_{U}+\lambda H+\left(2\lambda+\gamma\right) W-\left(2\gamma+\mu\right) U \end{matrix}$ (26)

The first equation represents population demographics with source $S_{H}=\sum_{i=0}^{\infty} S_{i}$and decay (turnover) rate$\mu$. The higher-moment equations (W, U) have sources

$\begin{matrix} S_{W}=\sum_{i=1}^{\infty} i\cdot S_{i}\geq0 \\ S_{U}=\sum_{i=1}^{\infty} i^{2}\cdot S_{i}\geq0 \end{matrix}$ (27)

determined by $\left\{S_{n}\right\}$. Clearly, both vanish ($S_{W}=S_{U}=0$) provided the original SWB (25) had only uninfected sources ($S_{k}=0$, for $k\geq1$), such as newborn recruits to the youngest-age cohort. The moment system (26) can be recast for mean variables: (MWB) and , with sources $S_{w}=S_{W}/H$ , $S_{u}=S_{U}/H$

$\begin{matrix} \frac{dw}{dt}=S_{w}+\lambda-\left(\gamma+\mu\right)w \\ \frac{du}{dt}=S_{u}+\lambda+\left(2\lambda+\gamma\right)w-\left(2\gamma+\mu\right)u \end{matrix}$ (28)

The human equations (28) can be coupled to the snail equation via force of infection , with a proper mating function , as in the standard MacDonald-MWB case. Assuming NB strata $\left\{h_{n}\left(t\right)\right\}$ the NB aggregation factor, k, can be expressed through mean *w* and variance (2^nd^ moment) *u* as

$k\left(w,u\right)=\frac{w^{2}}{u-w^{2}-w}$ (29)

We substitute it in the mating function (Appendix S3, equation (36)) of snail FOI, to get the snail equation coupled to (28)

(30)

Equilibrium solutions of (28) depend on 4 parameters: dimensionless$\left(\lambda,\mu\right)$ (rescaled over $\gamma$) and two demographic sources $\left(S_{w},S_{u}\right)$, namely

$\begin{matrix} w^{*}=\frac{\lambda+S_{w}}{1+\mu} \\ u^{*}=\frac{\lambda\left(2\lambda+2+\mu\right)+S_{w}\left(1+2\lambda\right)+S_{u}\left(1+\mu\right)}{\left(1+\mu\right)\left(2+\mu\right)} \end{matrix}$ (31)

Hence the equilibrium aggregation

$k^{*}\left(\lambda,\mu,S_{w},S_{u}\right)=\frac{(S_{w}+\lambda)^{2}(2+\mu)}{\lambda^{2}\mu+S_{u}(1+\mu)^{2}-S_{w}^{2}(2+\mu)-S_{w}(2\lambda+(1+\mu)^{2})}$ (32)

When demographic sources are dropped (the youngest age group) equilibrium relation are simplified to

$\left\{w^{*},u^{*},k^{*}\right\}=\left\{\frac{\lambda}{1+\mu}, \frac{\lambda\left(2+2\lambda+\mu\right)}{\left(1+\mu\right)\left(2+\mu\right)}, 1+\frac{2}{\mu}\right\}$ (33)

# Appendix S3: Mating function in Macdonald and SWB systems

The force of snail infection depends crucially on the number of mated females (couples) in human hosts. Nasell-Hirsch [17] and May [12] studied different patterns of worm mating and parasite distribution. For a host strata with *n* adult worms, assuming a binomial distribution of males-females (togetherness), the expected fraction of mated worms is given by $f_{n}=1-2^{-2k}\left(\begin{matrix} 2k \\ k \end{matrix}\right)$ if $n=2k$ is even, and the same for the following odd number, $n=2k+1$ (see [7], [11], ). The resulting mated couple count is

$\psi_{n}=\frac{n}{2}f_{n}=\frac{n}{2} \left[1-2^{-2m} \left(\begin{matrix} 2m \\ m \end{matrix}\right)\right]$ , with $m=\left[n/2\right]$ = integer part of $n/2$. (34)

This formula is independent of worm distribution $\left\{h_{n}\right\}$. Thus, in our paper we applied it for the SWB system to estimate the snail FOI (15). May [12] used (34) for specific distributions, NB or Poisson, to estimate the mating function , i.e. fraction of mated worms in a host population, defined as

Assuming an NB worm distribution $h_{n}=\alpha^{k}\frac{\Gamma\left(k+n\right)}{\Gamma\left(k\right)\Gamma\left(n+1\right)}\left(1-\alpha\right)^{n}$, with mean *w* and aggregation k (or probability $\alpha=\frac{k}{w+k}$ ) May derived the following integral form of

(35)

He also found other forms of in terms of special functions, in particular the hypergeometric function

Specifically,

(36)

In the limiting Poisson case ($k=\infty$) [17], formulae (35) turn into

(37)

expressed through a modified Bessel functions $I_{n}\left(z\right)$ of order n,

$I_{0}\left(z\right)=\sum_{n=0}^{\infty} \frac{\left(z/2\right)^{2n}}{\left(n!\right)^{2}}$ ; $I_{1}\left(z\right)=\frac{1}{z}\sum_{n=1}^{\infty} \frac{2n\left(z/2\right)^{2n}}{\left(n!\right)^{2}}$.

# Appendix S4: Equilibria of a coupled human SWB - snail system

Equilibrium snail equation for the SWB system (13) – (17) is given by function

$F_{SW}\left(y\right)=\frac{B }{\nu}\cdot\sum_{n\geq1} \psi\left(n \Delta w, q^{\Delta w}\right)\cdot h_{n}^{*}\left(\lambda,\mu\right)\cdot\left(1-y\right)-y$ (38)

Here $\lambda=\frac{A y}{\gamma\Delta w}$ is the force of human infection (proportional to snail prevalence y), $\mu=\mu/\gamma$- rescaled population turnover, $\psi\left(n,q\right)=\psi_{n}\left(1-q^{n}\right)$ mated couple count, and $h_{n}^{*}\left(\lambda,\mu\right)$ is the equilibrium SWB distribution for prescribed$\lambda,\mu$.

Similar to the Macdonald-MWB case (18), the conditions for “triple” equilibrium (bistable case) are (i) $\max\left[F_{SW}\left(y\right)\right]>0$, and (ii) slope $\frac{d}{dy}F_{SW}\left(0\right)<0$. The slope of $F_{SW}\left(y\right)$ at $y=0$ is computed from SWB transition matrix of system (21) $T\left(\lambda\right)=\lambda L+U$, expanded into the sum of upper (U) and lower (L) triangular matrices

$$L=\left[\begin{matrix} 1 & 0 & \cdots& 0 \\ -1 & 1 & 0 & \vdots\\ 0 & \ddots& \ddots& 0 \\ 0 & \cdots& -1 & 1 \end{matrix}\right]; U=\left[\begin{matrix} \mu& -1 & \cdots& 0 \\ 0 & \mu+1 & -2 & \vdots\\ \vdots& \ddots& \ddots& -n \\ 0 & \cdots& 0 & \mu+n \end{matrix}\right]$$

The equilibrium solution $\hat{h}\left(\lambda,\mu\right)=T\left(\lambda,\mu\right)^{-1}\cdot\hat{S}$, for the “young-age” source $\hat{S}=\left(\mu,0,...,0\right)$ , can be computed by inverse matrix applied to uninfected strata $\hat{h}\left(0,\mu\right)=\left\{1,0,0,...\right\}$ $\frac{d}{d\lambda}\hat{h}\left(0,\mu\right)=-U^{-1}\cdot L\cdot\hat{h}\left(0,\mu\right)$. Hence, the explicit relation

$\frac{d}{dy}F_{SW}\left(0\right)=\frac{A B}{\nu\cdot\left(\gamma+\mu\right)\Delta w} \psi\left(\Delta w,q^{\Delta w}\right)-1=\psi_{\Delta w}R_{0}-1$ (39)

where $R_{0}=\frac{A B}{2\left(\gamma+\mu\right)\nu}$ is the standard MacDonald BRN (18) adjusted for population turnover$\left(\gamma+\mu\right)$. The condition for stability of equilibrium zero is

$$R_{0}=\frac{A B}{2\left(\gamma+\mu\right)\nu}<\frac{1}{\psi_{\Delta w}}$$

It gives the upper bound of the (shaded) breakpoint region in Figure 6, its lower bound is given by the curve $\max_{y} \left[F\left(y,a,b,q\right)\right]=0$, computed numerically.

# Appendix S5: Model calibration

As a test case for model comparison, we take a simple setup made of a single host population with an uninfected source, and stationary demographics (fixed population size and turnover rate). A typical epidemiological and demographic data profile for such a system are shown in Table 4. The former includes population turnover (e.g. life-span), and known worm and snail mortality. The infection data typically comes from the egg-count diagnostics (*S. mansoni* eggs per gram (epg) feces by Kato-Katz smear or *S. haematobium* eggs per 10 mL urine by filtration). The table gives two measure of human infection: prevalence $0<P<1$ , and mean egg-count $E$, proportional to the mated-couple count ($E=\rho\Psi$) , where $\rho$ is (known) “mean epg production/release per mated couple”. Snail infection is measured by its prevalence $0<y^{*}<1$. The goal of model fitting is then to calibrate model parameters: transmission rates ($A,B,k$) for the MacDonald system, and ($A,B,q$) for the SWB system.

We assume the baseline endemic state of the system and use equilibrium equations (9) and (13) – (16) for calibration. Both systems predict the same transmission rate *B* estimated from “snail + mean EPG” data

$B=\frac{\nu y^{*}}{\left(1-y^{*}\right)E/\rho}$ . (40)

For rate *A* we get different expressions in the MacDonald (through equilibrium MWB $w^{*}$)

$A=\frac{\left(\gamma+\mu\right)w^{*}}{y^{*}}$ , (41)

and in the SWB case (in terms of the rescaled FOI $\lambda^{*}$ and strata increment $\Delta w$)

$A=\frac{\lambda^{*}\gamma\Delta w}{y^{*}}$ , (42)

To estimate $w^{*}$, or $\lambda^{*}$ we employ the human prevalence data along with egg-count $E/\rho$. Specifically, equilibrium MacDonald equations with NB-prevalence function $\Omega\left(w,k\right)$ and mating function (Table 2).

are solved for $\left(w,k\right)$ to get equilibrium values$\left(w^{*},k^{*}\right)$. A similar SWB equations with prevalence $\Omega\left(\lambda,\mu\right)$ of (8), and mated count $\Psi\left(\lambda,\mu,q\right)$ of (3),

$\left\{\begin{matrix} P=\Omega\left(\lambda,\mu\right) \\ E/\rho=\Psi\left(\lambda,\mu,q\right) \end{matrix}\right.$ (43)

are solved to get $\left(\lambda^{*},q^{*}\right)$. The calibration results for an author-selected, representative data set (Table 4) are given in Table 5.

Also, for comparison, we calibrated “simple” versions of both systems (MacDonald and SWB) without mating (). Simple models have fewer parameters than the infection data. So we used only mean egg-count $E/\rho$ (proportional to MWB) for “simple MacDonald”, and a combination of “simple SWB” prevalence ([23]) and its mated worm count, to fit a single parameter $\lambda$ to the chosen data. Calibrated transmission rates *A* proved to be different for all 4 systems, with the highest value attained for mated SWB, which indicates its lower estimated transmission potential compared to other models.

Let us note that for demonstration purposes we have chosen a data set that would give a breakpoint-type SWB system. Figure 8 shows two reduced equilibrium functions: MacDonald’s $F\left(y\right)$ of (19) (shown in gray), and the SWB-function $F_{SW}\left(y\right)$ of (21) (in black). Both exhibit breakpoints near *y=0*, but SWB has higher breakpoint value $y_{B}^{*}$ , than MacDonald’s (see Table 5). This suggests that a SWB infection system would be easier (faster) brought to elimination by MDA compared to the corresponding MacDonald-MWB systems.
