# Supplementary material for: Correction: Population Biology of Schistosoma Mating, Aggregation, and Transmission Breakpoints: More Reliable Model Analysis for the End-Game in Communities at Risk
Source: PLoS One. 2015 Apr 1;10(4):e0123592. doi: 10.1371/journal.pone.0123592 (PMC4382302; doi:10.1371/journal.pone.0123592)
Supplement: S1 File — (PDF) [file pone.0123592.s001.pdf]

PLOS ONE

RESEARCH ARTICLE

Population Biology of *Schistosoma* Mating, Aggregation, and Transmission Breakpoints: More Reliable Model Analysis for the End-Game in Communities at Risk

David Gurarie[1,2], **Charles H. King**[2]*

1. Department of Mathematics, Case Western Reserve University, Cleveland, Ohio, United States of America, **2.** Center for Global Health and Diseases, Case Western Reserve University, Cleveland, Ohio, United States of America

*chk@cwru.edu

CrossMark
click for updates

OPEN ACCESS

Citation: Gurarie D, King CH (2014) Population Biology of *Schistosoma* Mating, Aggregation, and Transmission Breakpoints: More Reliable Model Analysis for the End-Game in Communities at Risk. PLoS ONE 9(12): e115875. doi:10.1371/journal.pone.0115875

Editor: Ulrike G. Munderloh, University of Minnesota, United States of America

Received: August 13, 2014

Accepted: December 2, 2014

Published: December 30, 2014

Copyright: © 2014 Gurarie, King. This is an open-access article distributed under the terms of the Creative Commons Attribution License, which permits unrestricted use, distribution, and reproduction in any medium, provided the original author and source are credited.

Data Availability: The authors confirm that all data underlying the findings are fully available without restriction. All relevant data are within the paper.

Funding: D. Gurarie was supported by the sabbatical fellowship at NIMBioS (University of Tennessee) during Academic Year 2012–13. This work is also supported by the Schistosomiasis Consortium for Operational Research and Evaluation (SCORE) funded by the University of Georgia Research Foundation through a grant from the Bill and Melinda Gates Foundation. The funders had no role in study design, data collection and analysis, decision to publish, or preparation of the manuscript.

Competing Interests: The authors have declared that no competing interests exist.

Abstract

Mathematical modeling is widely used for predictive analysis of control options for infectious agents. Challenging problems arise for modeling host-parasite systems having complex life-cycles and transmission environments. Macroparasites, like *Schistosoma,* inhabit highly fragmented habitats that shape their reproductive success and distribution. Overdispersion and mating success are important factors to consider in modeling control options for such systems. Simpler models based on mean worm burden (MWB) formulations do not take these into account and overestimate transmission. Proposed MWB revisions have employed prescribed distributions and mating factor corrections to derive modified MWB models that have qualitatively different equilibria, including 'breakpoints' below which the parasite goes to extinction, suggesting the possibility of elimination via long-term mass-treatment control. Despite common use, no one has attempted to validate the scope and hypotheses underlying such MWB approaches. We conducted a systematic analysis of both the classical MWB and more recent ''stratified worm burden'' (SWB) modeling that accounts for mating and reproductive hurdles (Allee effect). Our analysis reveals some similarities, including breakpoints, between MWB and SWB, but also significant differences between the two types of model. We show the classic MWB has inherent inconsistencies, and propose SWB as a reliable alternative for projection of long-term control outcomes.

Introduction

In the last decade, greater recognition of the sub-clinical, but disabling effects of schistosomiasis has led to a new awareness of the importance of preventing *Schistosoma* infection and reinfection among populations at risk [1]. Because of a better understanding of the long-term consequences of the chronic inflammation triggered by anti-*Schistosoma* immunity [2, 3], it is no longer considered sufficient to provide just 'morbidity control' (via suppression of infection intensity), as has been advocated in the past [4, 5]. Instead, it has become a priority to find practical means to interrupt transmission and provide local elimination of infection wherever possible. This objective has been outlined in the 2012 London Declaration for Neglected Tropical Diseases (NTDs, http://unitingtocombatntds.org/resource/london-declaration) and in the World Health Organization's 2020 Roadmap on NTDs [6], and codified in World Health Assembly resolution 65.21. These initiatives now seek tools to aid the goal of local elimination of schistosomiasis. As a result, it seems appropriate to re-evaluate existing transmission models of dioecious macroparasites (like *Schistosoma*) for their usefulness in implementing effective policy in areas that will experience declining human and intermediate host infection prevalence under the pressure of current infection- and transmission-control interventions.

In 1965, the dynamic modeling of MacDonald [7] prompted hopes there could be an ecological 'breakpoint' in *Schistosoma* spp. transmission if the local numbers of intermediate host snails could be reduced by 90% or more [8–10]. This local extinction was projected as a consequence of the obligate need for sexual reproduction by the parasite (dioecy) within the human host; if male and female worms could not combine within the same host, then transmission was effectively ended. MacDonald's analysis projected that there would be special leverage in obtaining reductions in transmission by interventions that limit snail-to-human transmission [7]. Such reductions were seen to be potentially achievable by existing modalities of chemical mollusciciding and/or snail habitat destruction. Effectively, the breakpoints described in MacDonald's work [7] and subsequent studies [11, 12], are points or regions within the transmission parameter space below which existing worm burden is unsuccessful in maintaining transmission, and parasite numbers ultimately go to zero without further intervention.

Questions remain: do breakpoints exist in real world settings?, and does this phenomenon have relevance, *i.e.*, yield a practical benefit in the context of community-wide *Schistosoma* control campaigns? In practice, where prolonged and extensive reductions of snail numbers were achieved [8, 13–16], *Schistosoma* prevalence often dropped, but transmission was not interrupted, suggesting flaws in the basic assumptions in the MacDonald model [7, 17], and additional complicating factors with regard to parasite mating patterns [12, 18]. In addition, heterogeneities in water habitat distribution and in human water contact behavior [19, 20], were suspected to contribute strongly to the persistence of *Schistosoma* within suitable ecosystems.

Bradley and May [18] point out that the typically observed 'clumping' of high worm burdens among a small fraction of human hosts (overdispersion with aggregation) could lead, overall, to a lower stability of transmission. However, with greater aggregation, if male and female worms are transmitted in roughly equal numbers to each human host (or if worm mating is promiscuous and not monogamous) [21], then the projected breakpoint phenomenon might not prove relevant, because females are increasingly likely to be successfully mated in such scenarios. As a consequence, egg output, and hence human-to-snail transmission, will persist, albeit at lower levels. They note, however, that if the acquisition of male worms and of female worms is aggregated in separate fashion for each sex (as might occur with very low worm burdens in a low-transmission environment) then breakpoints are more likely to be relevant, and spontaneous failure of parasite transmission is more likely to occur.

To evaluate the likely relevance of the breakpoint phenomenon in current control efforts, the present study compares the projections of two established modeling approaches to *Schistosoma* transmission: i) the modified MacDonald-type Mean Worm Burden (MWB) model proposed by May and colleagues [12, 18] and by Nåsell and colleagues [11, 17] utilizing their assumed negative binomial (NB) or Poisson distributions of worm burden; and ii) a distribution-free Stratified Worm Burden (SWB) approach we have previously developed [22, 23], and now modify to include the effects of parasite mating probability (see Table 1 for a list of abbreviations used in this paper).

Of special interest is each model system's projections (and their limitations) when parasite burdens get very low. In brief, we find that the classical MWB approach and its extensions have shortcomings that limit their usefulness in projecting elimination in various transmission settings (see S1 Appendix in S1 Text,). In prior work [23], we have advocated the use of SWB systems as an improved alternative to the more analytically tractable (but potentially less realistic) MWB systems [24]. While SWB are high-dimensional models (depending on the number of strata), they can now be efficiently implemented, simulated, and studied numerically.

Our current approach allows a more detailed account of density-dependent mating factors–we are now able to include the so-called Allee effect (common for many species [25]), in which growth and subsequent mating success are significantly impaired when population numbers get very low in a given within-host environmental 'patch'. For schistosomes, we propose that the Allee effect obtains when female worms fail to mature in the absence of sufficient males [26], leading to disproportionately lower transmission despite persistent (albeit low) mean worm burden in human hosts. Additionally, we indicate how the SWB approach can be extended to accommodate highly influential geographic and age-related demographic factors [19, 20].

Table 1. Symbols and abbreviations used in this paper.

MWB	Mean Worm Burden
SWB	Stratified worm burden
NB	Negative binomial (distribution)
MDA	Mass drug administration
FOI	Force of infection
R_0BRN or	Basic reproduction number in MacDonald MWB system
$R_c(k,a)$	critical BRN-type parameter for breakpoint in MWB system
H	Total host population
h_n	n-th human strata in SWB system (population fraction carrying n worms)
S_n	Demographic sources for nth strata h_n in SWB system
Δw	Worm increment in SWB formulation
w	Worm burden (MWB), $w=\sum_{n=1}^{\infty} n\,h_n - 1^{st}$ moment of SWB system
u	$u=\sum_{n=1}^{\infty} n^2\,h_n 2^{nd}$ moment of SWB system:
W	Total worm population (=*H w*)
U	Total 2nd moment (=*H u*)
$S_w; S_u$	Source terms in the w- and u- equations
k	NB (negative binomial) aggregation parameter
μ	Human population turnover rate (.02−.05/year)
γ	Worm mortality rate (=1/4 years)
v	Snail mortality (=5/year)
λ	Human FOI (=mean rate of adult worm accumulation in human hosts)
Λ	Snail FOI (transition rate ‘‘susceptible’’ −> ‘‘infected’’)
$\theta(i,j)$	Mated female number for mixed strata (*i* males and *j* females)
ψ_n	Mated female count for n-th strata made of *n* adult worms
Ψ	Total mated female count in host population
$\phi=\phi(w,k)$	Mating function in MacDonald (MWB) system with NB worm distribution
q	Allee mating hurdle factor ($0<q<1$)
A	Transmission rates (snail->human) per single infected snail
B	Transmission rates (human->snail) per mated female
$a=\frac{A}{\gamma};\ b=\frac{B}{v}$	Relative transmission rates
$\Omega(w,k)$	Infectious prevalence in MWB system with mean *w*, and NB aggregation *k*
$\Omega(\lambda,\gamma)$	μInfectious prevalence in SWB system with FOI λ, and host turnover
$F(y,\ldots)$	Reduced ‘‘snail equilibrium’’ function for MWB (MacDonald) system
$F_{SW}(y,\ldots)$	Reduced ‘‘snail equilibrium’’ function for SWB system
	Drug efficacy (fraction of worms surviving a single dose)
f	Population fraction cover in MDA
E	Mean egg count in host population
ρ	Egg production/mated female
$y^*; w^*; \lambda^*; h_n^*$	Equilibrium levels for MWB, SWB systems

doi:10.1371/journal.pone.0115875.t001

Methods

Modeling worm aggregation in MWB and SWB

Different approaches have been used to describe *Schistosoma* worm distributions, ranging from a nearly uniform host burden (whereby each host carries approximately $w = W/H$ worms- the total parasite load W distributed evenly over host population – H), to types of over-dispersed distribution such as the negative binomial (NB) distribution or the Poisson distribution. The former, NB, is defined by two parameters: aggregation, k, and mean w, where probability $\alpha = k/(k+w)$ and

$$h_n(w,k) = \alpha^k \binom{n+k-1}{k-1} (1-\alpha)^n, \quad n = 0,1,2,... \tag{1}$$

Increased k gives a more aggregated (clumped) distribution for $\{h_n(w,k)\}$; in the limiting case where $k = \infty$, the NB distribution becomes the Poisson distribution.

Overdispersed parasite burden has been noted in many wildlife populations, and the NB has been proposed as the best model for this phenomenon [27–29]. In most cases, the apparent aggregation factor was relatively low, but found to vary widely among different species and locations. Despite its resemblance to empirical data, there is no biological dictate for choosing the NB distribution based on first principles. A multitude of biological, environmental and other factors can affect parasite distributions within definitive hosts, and the only justifiable pattern derived from the underlying principles (random worm acquisition), has been the Poisson case advocated by Nåsell & Hirsch [17].

The NB assumption has been widely used in modeling studies of macroparasite transmission [7, 11, 12, 29–31]. In these works, a prescribed distribution of worm burden (NB or Poisson), has been used to reduce a large (infinite-dimensional) stratified system of $\{h_n\}$ to a low-dimensional (MacDonald-type) "moment system" for the MWB variable $w(t)$, and/or higher moments (reviewed in S2 Appendix in S1 Text). The reduced models, unlike the SWB [23], can be analyzed mathematically [24]. On the other hand, the SWB approach requires no *a priori* assumptions on distribution or aggregation of strata $\{h_n\}$. Both SWB values arise naturally from the underlying processes of worm acquisition and loss. In future, our very practical interest will be to apply the calibrated SWB systems to demographically- or geographically-structured populations resembling problem areas that confront elimination program planners [32].

For such a complex, extended community, each population group can be represented by its own SWB system (Fig. 1), and these separate systems coupled via suitable source parameters. In Fig. 2, SWB equilibrium distributions are compared to the standard NB/Poisson case. The simplest 'single population' SWB system (Fig. 1) has three main parameters: λ, human FOI, balanced by worm mortality, γ, and demographic loss, μ. Its equilibrium distribution $\{h_n(\lambda',\mu')\}$ depends on rescaled values $\lambda' = \lambda/\gamma$ and $\mu' = \mu/\gamma$, the former λ' having dimension

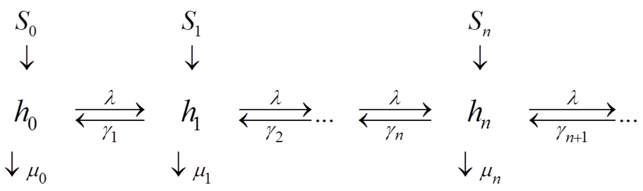

Fig. 1. Schematic diagram of a Stratified Worm Burden (SWB) system. The SWB model includes population strata $\{h_n(t)\}$, sources $\{S_n\}$, population turnover/loss rates $\{\mu_n\}$, the force of infection λ (=worm accretion rate), and worm clearing rates $\gamma_n = n\gamma$ (γ is worm mortality).

doi:10.1371/journal.pone.0115875.g001

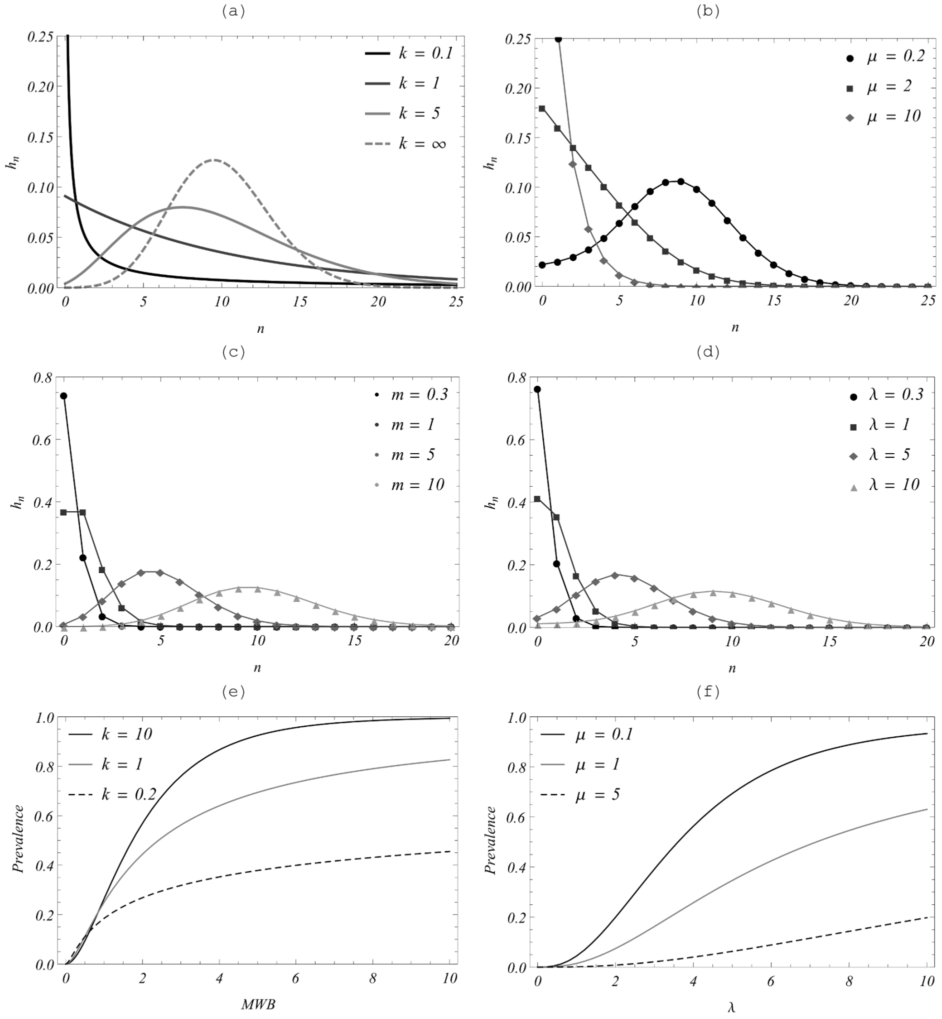

Fig. 2. Comparison of worm distribution patterns for a negative binomial-based MWB system *vs.* a SWB system. Here, all SWB distributions (right-hand panels) are produced from an uninfected source $\{S_n\}$. (a) NB-MWB with fixed mean $w = 10$ and increasing k ($k = \infty$ is the limiting Poisson case); (b) Equilibrium SWB distribution for uninfected source with FOI (mean) $\lambda = 10$, and varying demographic parameter μ (see equation (13)); μ plays the role of aggregation k for NB, with small μ corresponding to large (infinite) k. Panels (c−d) Poisson distributions with different means, m_i (left panel) *vs.* SWB distributions with different $\lambda_i = m_i$ (right panel) exhibit striking similarity. Panels (e)−(f) compare infectious prevalence Ω for the two models as a function of NB-MWB *w* or SWB λ.

doi:10.1371/journal.pone.0115875.g002

of [worm burden] like the MacDonald-MWB term w, while μ' is dimensionless. The resulting SWB solutions depend on demographic source terms (equations (13) below).

For better comparison to the basic NB/Poisson distributed models, we can use a simplified SWB system with only an uninfected source, *i.e.,* a source term adding to the uninfected strata, h_0, only ($S_0>0$, $S_n=0$ for n>0). Such sources would obtain in closed (isolated) populations, and be relevant to the youngest age-group (a newborn source) in the studied population. In general, equilibrium SWB distributions have no analytic formulae, so most results below are computed numerically. The only analytically tractable case corresponds to the limiting (degenerate) system, $\mu=0$, $S=0$ (no population turnover and sources). Here equilibrium solution gives the standard Poisson distribution $h_n(\lambda,0)=\frac{e^{-\lambda}\lambda^n}{n!}$, consistent with Nåsell & Hirsch [17].

Adding mating patterns as functions in the extended MWB and SWB

A simple way to account for mating behavior of adult worms is via a pairing function $\theta(i,j)$=the number of mated (egg-shedding) females for i - males, j - females. Several studies have looked the effect of mating on snail infection in the MWB type models [11, 12]. Examples for possible types of mating included:

$$\theta(i,j)=\begin{cases}\min(i,j) & \text{- monogamous mating}\\ \min(q\,i,j) & \text{- polygamous ("q-females" per "male")}\\ \min(n-1,j) & \text{promiscuous}\end{cases}$$

The mating patterns will affect transmission by determining the number of mated females, their effective egg production, and, as a consequence, the resulting force of snail infection, Λ. Specifically, for a given sex distribution $P(i,j|n)$ in the n-th stratum h_n $(i+j=n)$, the expected number of fertilized females is given by

$$\psi_n=\sum_{i+j=n}P(i,j|n)\cdot\theta(i,j), \quad (2)$$

Hence their mean (or total) number in host population

$$\Psi=\langle\psi_n\rangle=\sum_{n=0}^{\infty}\psi_n h_n \quad (3)$$

The force of snail infection $\Lambda = B \cdot \Psi$ is proportional to Ψ, with the transmission rate/worm, B, dependent on multiple factors including egg production/release per female, intermediate larval survival in the transmission environment, and human host behavior.

To compute Ψ and Λ, one needs some assumptions on *worm acquisition*, the sex ratio distribution $P(i, j|n)$, the mating pattern θ(above), and other fecundity/fitness parameters. May [12] distinguished two types of worm acquisition:

(i) *Togetherness* whereby both sexes come through the same accumulation process with equal probability (=0.5), where the sex ratio obeys a standard binomial:

$$P(i, j|n) = 2^{-n}\binom{n}{i} \tag{4}$$

(ii) *Separateness* whereby each sex comes from its own (independent) accumulation process, hence

$$P(i, j|n) = P(i|n)P(j|n) \tag{5}$$

For the present, field data on *Schistosoma* infection in rats [29] suggest that *togetherness* is the proper mating pattern in the wild. Using equation (4) leads to a closed form expression for the mated female count

$$\psi_n = \frac{n}{2}\left[1 - 2^{-2m}\binom{2m}{m}\right] \tag{6}$$

with $m = [n/2]$ - "integer part" of $n/2$ (see [12] and S3 Appendix in S1 Text for details).

Function $\{\psi_n\}$ can now enter our SWB formulation of Λ (as equation (3)). It was used with prescribed (NB, Poisson) distribution patterns, $\{h_n\}$ in earlier works [7, 12] to derive a suitable *mating function* $0 < \phi(w) < 1$. The latter measures the expected fraction (probability) of mated females per host, and the total mated (female) count expressed as:

$$\Psi = \frac{w}{2}\phi(w) \tag{7}$$

In special cases of distribution $\{h_n\}$ (uniform burden, Poisson, or NB) the mated female worm count Ψ, and mating function ϕ can be computed in closed analytic form (see Table 2, Fig. 3, and, for derivation, S3 Appendix in S1 Text).

A parameter commonly used to define the efficacy of transmission control is the *infectious* prevalence, Ω, defined as the population fraction that carries at least one *mated couple* (different from the commonly used *infection* prevalence based

Table 2. Mating function for specific distribution patterns; ${}_2F_1(a, b, c|z)$ is the hypergeometric function, $I_m(z)$ - modified Bessel functions of order m.

Distribution	Mating function	Prevalence of mated couples (host infectivity) Ω
Uniform with mean *w*	$\phi(w)=1-2^{-w}\frac{\Gamma(w+1)}{\Gamma(w/2+1)^2}$	
Poisson with mean *w*	$\phi(w)=1-e^{-w}[I_0(w)+I_1(w)]$	$\left(1-e^{-w/2}\right)^2$
NB of mean *w*, aggregation *k*	$\phi(w,k)=1-{}_2F_1\left(\frac{1}{2},1+k,2\middle|-\frac{2w}{k}\right)$	$1-2\left(1+\frac{w}{2k}\right)^{-k}+\left(1+\frac{w}{k}\right)^{-k}$

(*abbreviation:* NB, negative binomial, see S3 Appendix in S1 Text for details).

doi:10.1371/journal.pone.0115875.t002

on worm count). To estimate Ω we note that the probability of "zero couples" in the n-th strata is $1-2^{1-n}$. Hence for SWB formulation,

$$\Omega=\sum_{n=2}^{\infty}\left(1-2^{1-n}\right)h_n \qquad (8)$$

For MacDonald-MWB type systems with prescribed distribution (NB, Poisson), May [12] derived explicit formulae for Ω (Table 2). Therefore, in our revised estimation of community transmission, we may now include the function Ω in calibrating model parameters based on diagnostic egg count data from control programs.

Numeric implementation

Our analysis of MWB and SWB systems combines analytic tools with a substantial amount of numeric simulations. The latter applies to equilibria and parameter

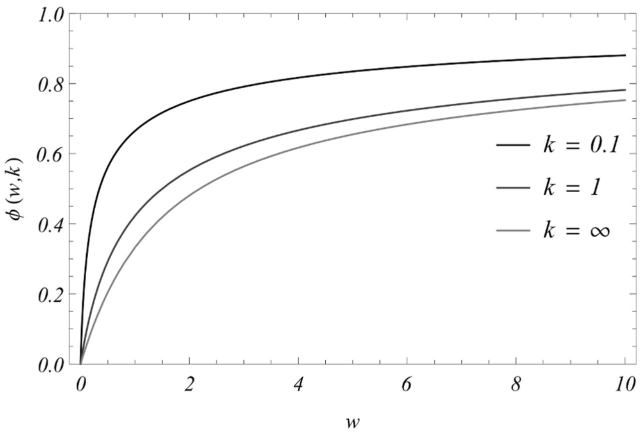

Fig. 3. Mating function $\phi(w,k)$ (Table 2) for increased negative binomial aggregation values. Shown are results for $k=.1;\ 1;\ \infty$ (Poisson). Note that a higher degree of clumping lowers $\phi(w,k)$ and reduces transmission potential of the system.

doi:10.1371/journal.pone.0115875.g003

space analysis on the one hand, and to dynamic simulations for prediction/control on the other. All numeric codes and procedures were implemented and run in Wolfram Mathematica 9, with differential equation solvers that offer event-control tools adapted for simulation and analysis of control interventions. A basic version of our Mathematica program notebook (nb) for this analysis is posted online as S1 Workbook, to this paper.

Transmission Models that Are Compared

The Macdonald MWB system

This simpler model of *Schistosoma* transmission for a single population has two variables: $w(t)$- the MWB of host population, and patent (or infectious) snail prevalence, $y(t)$. When including a mating factor, these variables obey a coupled differential system:

$$\begin{aligned}\frac{dw}{dt}&=A\cdot y-\gamma w\\ \frac{dy}{dt}&=B\cdot\phi(w)\ \frac{w}{2}\ (1-y)-\nu y\end{aligned}\tag{9}$$

The mating function $0<\phi(w)\leq 1$ depends on underlying assumptions on parasite distribution (NB, Poisson, etc.) and, in many cases, it can be computed explicitly (see Table 2, Fig. 3, and S3 Appendix in S1 Text).

Transmission rates A and B lump together multiple biological, environmental, and human behavioral factors and reflect the success of intermediate larval stages. In particular, A is proportional to snail population density (N), $A=a\,N$ (with per capita rate$=a$), while B is proportional to total human population (H), $B=b\,H$. Equation system (9) assumes stationary values for (H, N), but it can be easily extended to non-stationary cases (*e.g.,* changing human demographics, or seasonal variability of snail population and transmission). One can also include the population turnover (rate μ) in equations (9), by changing worm loss term $\gamma\rightarrow\gamma+\mu$ in the w-equation. The basic model can be further extended to various heterogeneous settings (age-structured and/or spatially distributed communities). Such modified MWB systems have been used extensively in the prediction/control analysis (see, *e.g.,* [20, 22, 33]).

The extended MacDonald MWB systems: moment equations and dynamic aggregation

A serious drawback of using the NB assumption in MacDonald-MWB systems is the uncertainty about (or evident variability) of the aggregation parameter k across time, age groups, and communities. This applies to different geographic environments and, more importantly, to the same system subjected to dynamic changes (*e.g.,* by drug treatment) [29]. So, fixing k, as estimated from observed data, in the mating function $\phi(w, k)$ of equations (9) leads to inconsistency, as

shown in drug treatment simulation studies [34–36]. To accommodate changing k values, some workers have proposed making k a dynamic variable, *e.g.*, $k = k_0 + k_1 w$ - a linear function of w, and then estimating coefficients (k_0, k_1) from field data [35, 36]. In general, one could expect an increase of k with w (a higher average burden implies higher aggregation), but, in reality, the relationship may not be linear.

A more consistent way to introduce dynamic aggregation within the NB-framework is via moment equations derived from the underlying SWB, namely 1st moment (MWB) - $w(t) = \sum_1^\infty i \cdot h_i(t)$; 2nd moment - $u(t) = \sum_1^\infty i^2 h_i(t)$, etc. The resulting two-moment systems

$$\begin{aligned} \frac{dw}{dt} &= S_w + \lambda - (\gamma + \mu) w \\ \frac{du}{dt} &= S_u + \lambda + (2\lambda + \gamma) w - (2\gamma + \mu) u \end{aligned} \tag{10}$$

have human FOI, $\lambda = A\,y$, decay rates (γ - worm mortality, μ - population turnover), and the external (demographic) sources (S_w, S_u) derived from the underlying SWB source $\{S_n\}$ (see S2 Appendix in S1 Text). The moment system (10) for variables $\{w(t), u(t)\}$ can be coupled to snail equation as in (9) via mating function $\phi(w, k)$. Then the NB assumption on host strata gives an algebraic formula for aggregation k expressed through variables (w, u) as

$$k = \frac{w^2}{u - w^2 - w} \tag{11}$$

So the snail equation in (9) turns into

$$\frac{dy}{dt} = B \cdot \phi(w, k(w, u))\ \frac{w}{2}\ (1 - y) - v\, y \tag{12}$$

(see [37, 38], and S2 Appendix in S1 Text).

The SWB system

The stratified worm burden (SWB) approach has been used before in theoretical studies (*e.g.*, [33, 37, 38]), mostly to derive the reduced (MWB-type) "moment" equations, like (9) or (10)–(12), above. The basic dynamic variables of the SWB-system are population strata $\{h_n(t) : n = 0,1,\ldots\}$ with total population $H(t) = \sum_{i=0}^{\infty} h_i$ (schematic diagram shown in Fig. 1). They obey a differential equation system ([37–39])

$$\begin{cases} \frac{dh_0}{dt} = -(\lambda+\mu)h_0 + \gamma_1 h_1 + S_0; \\ \ldots\ldots \\ \frac{dh_i}{dt} = \lambda h_{i-1} - (\lambda+\gamma_i+\mu)h_i + \gamma_{i+1}h_{i+1} + S_i; \\ \ldots\ldots \\ \frac{dh_n}{dt} = \lambda h_{n-1} - (\gamma_n+\mu)h_n + S_n; \end{cases} \quad (13)$$

Here λ is per capita force of human infection (rate of worm accretion), μ - population turnover (combined mortality, aging, migration), $\gamma_k = k\gamma$ - resolution rate for k-th strata (proportional to worm mortality, γ), and $\{S_k\}$ - demographic source term. The latter represent infections brought into a given population group from outside. Thus the youngest age-group has only a newly born (uninfected) source $S_0 > 0$ (proportional to the birth rate), while all other $S_n = 0$. The older groups have their sources coming from younger groups, while in- and out-migration can also create additional sources and sinks for geographically coupled populations. The human part of the system (13) is coupled to the snail equation by two FOI factors: the human, $\lambda = A \cdot y$, and the snail, $\Lambda = B \cdot \Psi$, *i.e.*, proportional to the mated worm count given by function Ψ of equation (3).

The worm strata in the SWB setup are defined by a *worm increment* $\Delta w = 1, 2, \ldots$ per stratum, so h_n consists of hosts carrying $n\,\Delta w \le w < (n+1)\Delta w$ worms. In theoretical studies, fine-grain strata ($\Delta w = 1$) are commonly used, but for practical modeling applications, larger increments are more appropriate (see [23, 32]).

To extend the basic SWB setup [23, 32] by including parasite mating in the force of snail infection, Λ, we can use May's estimate (equation (6)) of the expected number of mated couples ψ_n (see [12] and S3 Appendix in S1 Text), but these estimates employ the optimal (combinatorial) male-female pairing count. Not all such couples are likely to be realized at low parasite densities, when the maturational (trophic) effects of male-female worm pairing may go missed [26]. To account for a low-density mating hurdle (the Allee effect [25, 26]), we augment May's factors $\{\psi_n\}$ with an additional *density-dependent success rates,* $1 - q^n$, where parameter $0 < q < 1$ - measures the probability of mating failure. This factor would predict lower mating success in low- n strata, but approach a higher mating success rate of 1 at higher n. The resulting count of mated pairs takes the form:

$$\psi(m, q) = \psi_m (1 - q^m) \quad (14)$$

The force of snail infection is now expressed as a combination of variables $\{h_m(t)\}$ with weights $\psi(m, q)$,

Table 3. Comparison of Mean Worm Burden and Stratified Worm Burden models: variables, equations, and parameters.

	MacDonald-type Mean Worm Burden System	Stratified Worm Burden System
Mean Burden	*w(t)*	$\Delta w \sum_{m=0}^{n} m h_m(t)$
Mated-pair count	$\frac{w}{2}\phi(w, k)$	$\Psi = \sum_{n\geq 0} \psi(n\,\Delta w) h_n$
Human Force of Infection	$\lambda_{Md} = A \cdot y$	$\lambda_{SW} = \frac{A \cdot y}{\Delta w}$
Snail Force of Infection	$\Lambda_{Md} = B \cdot \frac{w}{2}\phi(w, k)$	$\Lambda_{SW} = B \cdot \sum_{n\geq 0} \psi(n\,\Delta w) h_n$
Human equations	$\frac{dw}{dt} = \lambda_{Md} - (\gamma + \mu + H'/H) w$	$\frac{dh_m}{dt} = \lambda \cdot h_{m-1} - (\lambda + \mu + m\gamma) \cdot h_m + (m+1)\gamma \cdot h_{m+1} + S_m$
Snail equations	$\frac{dy}{dt} = \Lambda_{Md}(1-y) - vy$	$\frac{dy}{dt} = \Lambda_{SW}(1-y) - vy$

doi:10.1371/journal.pone.0115875.t003

$$\Lambda = B \cdot \sum_{m=1}^{n} \psi(m, q) h_m \tag{15}$$

Similar to MacDonald-MWB system's equations (9), the coupled SWB-snail system consists of the human part (equation (13)) with FOI $\lambda = A\,y$, and snail equation

$$\frac{dy}{dt} = \Lambda(1-y) - v\,y \tag{16}$$

Both human and snail equations need some modification when worm increment $\Delta w > 1$. Here FOI of equation (15) is changed into

$$\Lambda = B \cdot \sum_{m=1}^{n/\Delta w} \psi\left(m\,\Delta w,\, q^{\Delta w}\right) h_m \tag{17}$$

while the human FOI of equation (13) is changed from $\lambda = A\,y$ (at $\Delta w = 1$) to $\lambda = A\,y/\Delta w$.

It is important to elaborate the parallels and differences between the two types of models, and how this influences their predictions for transmission control. Table 3 summarizes the system components and formulae for the MWB and SWB modeling approaches. The key inputs for both cases are: μ - host turnover, γ - worm mortality, v - snail mortality. Some can be estimated from published studies (*e.g.*, $\gamma = .25$/year, $v = 4$–5/year, (see Table 4)), while others involve known demographics in endemic areas (*e.g.*, $\mu = 1/15$ year for children, etc.). The most important parameters are transmission rates *A*, *B*, which must be estimated from observed human and snail infection data.

Table 4. Data inputs used for model calibration.

Demographic		Infection	
Host turnover	$\mu = .025$/year	Human prevalence	$P = .19$
Worm mortality	$\gamma = .25$/year	Mated couple (based on mean EPG)	$E/\rho = .46$
Snail mortality	$v = 5$/year	Snail prevalence	$y^* = .6$

(*abbreviation:* EPG, *Schistosoma* eggs per gram feces).

doi:10.1371/journal.pone.0115875.t004

Results

MWB equilibria and breakpoints

The simplest MacDonald-MWB system (equations (9)), without a mating component (*i.e.*, $\phi = 1$) has a stable-unstable pair of equilibria (infection-free + endemic state), provided that the Basic Reproductive Number (BRN, also known as R_0)

$$R_0 = \frac{A\ B}{2\gamma\ v} > 1 \tag{18}$$

For $R_0 < 1$ it goes to elimination (stable zero equilibrium). The BRN R_0 is made of two factors that measure relative input of snail-to-human transmission ($T_{SH} = A/\gamma$), and human-to-snail transmission ($T_{HS} = B/v$). The former, T_{SH}, can be viewed as the maximal MWB-level (for a given transmission system) attained at the highest snail prevalence, $y^* = 1$. As mentioned, to account for population turnover, formula (18) should be modified by changing $\gamma \rightarrow \gamma + \mu$. It is important to note that, in this model, any system with $R_0 > 1$ cannot go to elimination, as any positive infection level (no matter how small) is predicted to eventually bring it back to the stable endemic state.

Addition of a mating function, $0 < \phi(w) < 1$, vanishing at w=0, has profound effect on equilibria and the dynamics of MWB equation (9) by turning it into a bistable system, [12, 39]. Now the transition from "stable zero" to a bistable (endemic) regime requires higher transmission rates. Specifically, there exists a critical value, $R_c = R_c(k,a) > 1$, depending on aggregation, k, and snail-to-human transmission $a = A/\gamma$, such that $R_0 > R_c$ is bistable (endemic), while $R_0 < R_c$ goes to elimination. Function $R_c(k,a)$ has no simple algebraic form like equation (18), but we can explore it numerically.

The analysis exploits the reduced snail equation

$$F(y;\ R_0,\ a,\ k) = y[R_0 \cdot \phi(a\,y,\ k) \cdot (1 - y) - 1] = 0 \tag{19}$$

obtained from equilibrated worm burden $w^* = \frac{A\,y}{\gamma}$ of system (9), so roots of F give equilibrium values y^*. Function, $F(y)$, has a typical S-shaped pattern over $0 \le y \le 1$, with either a single root $y = 0$, or triple root $0 < y_b < y^*$ (zero,

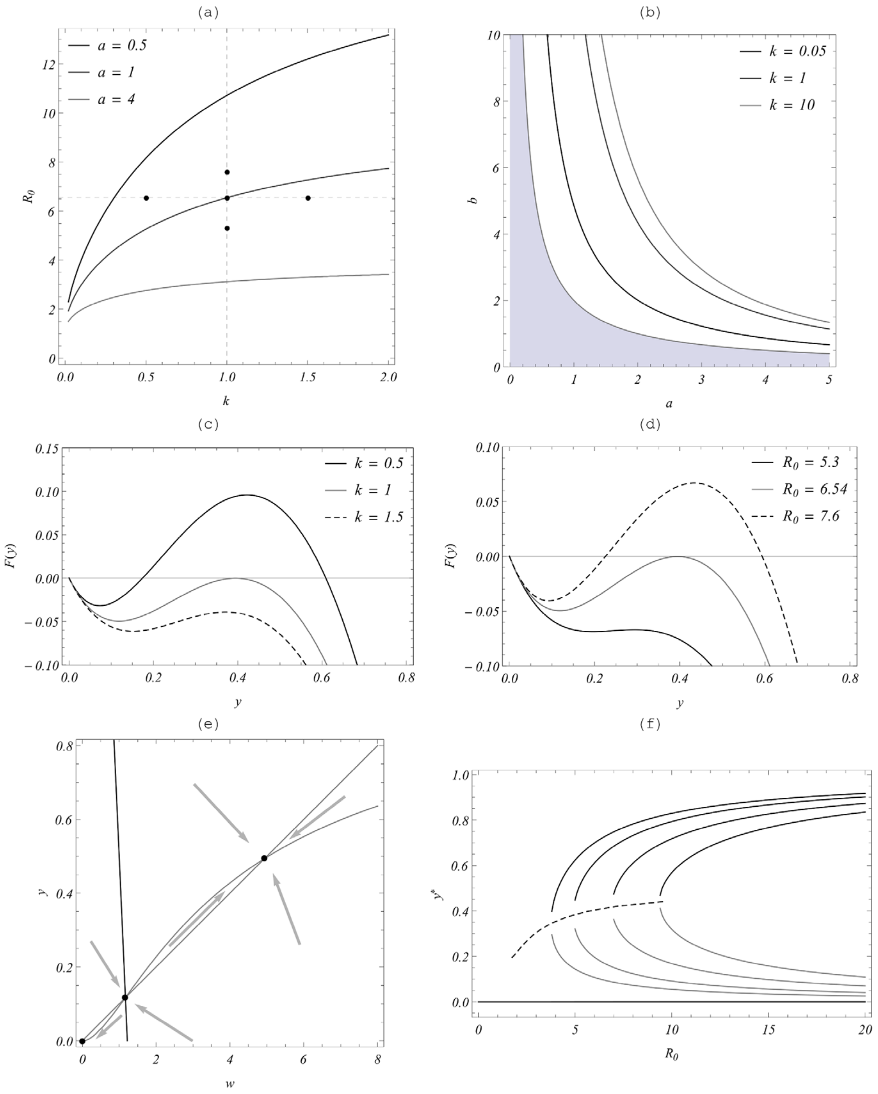

Fig. 4. Equilibria and stability regions of the Macdonald MWB system with parameters (a, b, R_0, k)**.** Panel (a) shows 3 critical (bifurcation) curves $R_c(k, a)$ in the (k, R_0) plane for $a = .5;\ 1;\ 4$. The region below each curve $R_0 < R_c(k, a)$ has stable zero (elimination), whereas the region above $R_0 > R_c(k, a)$ has a bistable (triple equilibrium) state. Panel (b) shows the same ''stable/unstable'' critical partition in the (*a*,*b*) parameter plane for three aggregation values $.05 < k < 10$. Panel (c) shows functions $F(y;\ R_0, a, k)$ of equation (19) for fixed $R_0 = 6.54$ $(= R_c(1,1))$, and $k = .4;\ 1;\ 1.5$ (above, at, and below critical R_c) corresponding to the three marked points on panel (a). Panel (d) shows functions $F(y;\ R_0, a, k)$ at fixed $k = 1$ and 3 values R_0 (above, at, below 6.54) marked on panel (a)). Panel (e) shows the phase plane of a bistable MWB system with three marked equilibria. Two ''separatrices'' at the breakpoint (in the middle) divide the phase plane into two attractor regions: ''zero'' - on the left and ''endemic'' - on the right. Panel (f) shows the bifurcation diagram of the MWB system for three types of equilibria, $y^*(R_0, a, k)$, where the upper branch is stable ''endemic'', the low is stable ''zero'', the middle (gray) are breakpoints. The dashed curve corresponds to critical (bifurcation) values $R_c(a, k)$. Here k=10, and the four curves arise from four different values of *a* in the range $1 \le a \le 6$ (increased *a* pushes bifurcation curves to the left).

doi:10.1371/journal.pone.0115875.g004

breakpoint, endemic), provided R_0 is sufficiently large, $R_0 > R_c$- critical (bifurcation) value. These features are demonstrated in Fig. 4.

Roots of $F(y)$ depend on three parameters: $\left(a = \frac{A}{\gamma}, R_0, k\right)$, or (a, b, k). As these parameters change, the system undergoes a transition from a stable "zero" state (single equilibrium) to a triple equilibrium state, illustrated in bifurcation diagrams of Fig. 4 (f).

(a) shows (k, R_0)- parameter plane separated by the critical $R_c(k, a)$ into "infection-free" range (below each curve) and "endemic/bistable" range (above it). In particular, values $k = 1$, $a = 1$, correspond to an $R_c = 6.54$. Panel (b) of Fig. 4 shows a similar "zero-endemic" partition in the parameter space ($a = A/\gamma$, $b = B/v$) for different levels of aggregation, k. As in panel (a), the respective infection-free ranges lie below the marked curves ($b < \frac{2}{a} R_c(k, a)$), and the endemic ranges above them. For comparison we also show in (b) the "infection-free" range ($\frac{a\,b}{2} \leq 1$) of a simple MacDonald-MWB system without mating (shaded). As shown, the effect of obligate mating (function $\phi(w, k)$) is to raise the thresholds for endemicity, $R_c(k, a) > 1$, and higher k (clumping) gives higher R_c values.

These results indicate that, under similar environmental conditions, sustained transmission in the "mated" MWB system is less likely that in the "simple" MWB, hence it would be easier to eradicate. Among different "k - systems" (NB vs. Poisson) higher clumping k makes the endemic state less tenable, and eradication potentially easier.

Panels 4(c) and 4(d) illustrate profiles of function $F(y)$ above and below bifurcation value R_c for several levels of aggregation (k), and BRN (R_0) values. Panel 4(e) shows a typical (w, y) phase portrait for a bistable MacDonald system with three equilibria, and schematic trajectories (arrows). The saddle-type breakpoint equilibrium (in the middle) has two "separatrices" (stable orbits) that divide the phase plane into two attractor regions: one solution driven to zero (elimination) on the left, and those relaxing to the endemic state on the right. Another effect of mating function $\phi(w, k)$ displayed in panels 4c, d, and f is a finite jump of endemic equilibria (w^*, y^*) as R_0 moves past the bifurcation value $(R_0 > R_c)$, typical for many bistable systems. In contrast, a simple $(\phi = 1)$ MWB endemic equilibrium undergoes a gradual transition $(y^* = 1 - 1/R_0)$ with R_0. This feature is related to *hysteresis*, whereby a gradual change of model transmission rates could, at a specific point, bring about a significant jump of endemic levels (an outbreak), rather than slow change.

In sum, sustained infection in any MacDonald-type system can be interrupted (brought to local extinction) by reducing transmission rates A, B (or $R_0 = A{\cdot}B/v{\cdot}\gamma$) below critical levels. But a typical intervention (MDA or snail control) does not affect the core transmission environment reflected by (A, B). Mathematically, a simple "no-mating" MacDonald system with BRN>1 cannot be brought to extinction, even after many control steps.

If valid, the breakpoint phenomena demonstrated for the extended MWB with mating in Fig. 4 could have significant implications for *Schistosoma* control [7, 12, 40]. The impact of control interventions are explored for MacDonald and SWB systems below.

The extended MWB system (10)–(12) can be analyzed similarly to the basic MWB case. Equilibria (w^*, u^*, k^*) of system (10) can be computed in terms of rescaled rates (λ, μ) (relative to γ), and demographic sources (S_w, S_u) contributed by birth, aging, and migration (for details see S2 Appendix's formulae (32)–(33) in S1 Text). Depending on population type (*e.g.*, a young cohort), (S_w, S_u) could be zero or nonzero.

Of special note, our analysis revealed substantial differences in predictions between the zero and nonzero source conditions. In the "zero" source case, equations (32) from S2 Appendix in S1 Text, give equilibrium value $k^* = 1 + 2/\mu$, independent of the transmission intensity λ, and indicate a k greater than 1, whereas observed aggregation values are often<1. The "nonzero" (positive) source case give a more complicated expression for k^* that can be studied numerically. The problem arises when function $k^*(\lambda, \mu, S_w, S_u)$ turns negative (non-physical) in certain regions of the (λ, μ)- parameter space. Such regions always exist, as long as $S_w, S_u > 0$. While demographic parameter μ is typically fixed, FOI λ(proportional to snail infection y) could undergo big changes due to interventions (MDA) that could take it into an "unphysical" domain. The only way to maintain strictly positive k^* throughout the (λ, μ) plane is to have zero source terms $(S_w = S_u = 0)$. The unphysical (λ, μ)-regions create problems for dynamic simulations in situations after MDA when λ changes abruptly, if computed results fall into unreal/impossible ranges.

SWB equilibria and breakpoints

A complete SWB system consists of an infinite set of variables $\{h_n(t)\}$ $(n = 0,1,2,\ldots)$, but for practical applications and computation, we truncate it at a finite (maximal) burden level N $(0 \le n \le N)$. The choice of N has minor effect on the system's behavior and outputs, as long as FOI λ (or MWB $w \approx \lambda$) remain small relative to N. Another practical consideration concerns SWB-granularity, defined by using worm increments $\Delta w \ge 1$. In some applications, it might be advantageous to reduce the number of variables (system dimensionality) by coarse-graining, from N $(\Delta w = 1)$ to $(N/\Delta w)$. Overall, an increased step Δw would lower FOI, Λ, but when done consistently, its effect could be minimized (see Fig. 5).

Another SWB input –mating hurdle $0 < q < 1$, related to the Allee phenomenon, has a more pronounced effect on model projections, particularly at low burden (small n). One way to assess it is through an estimated "mated count per worm" in the n-th strata,

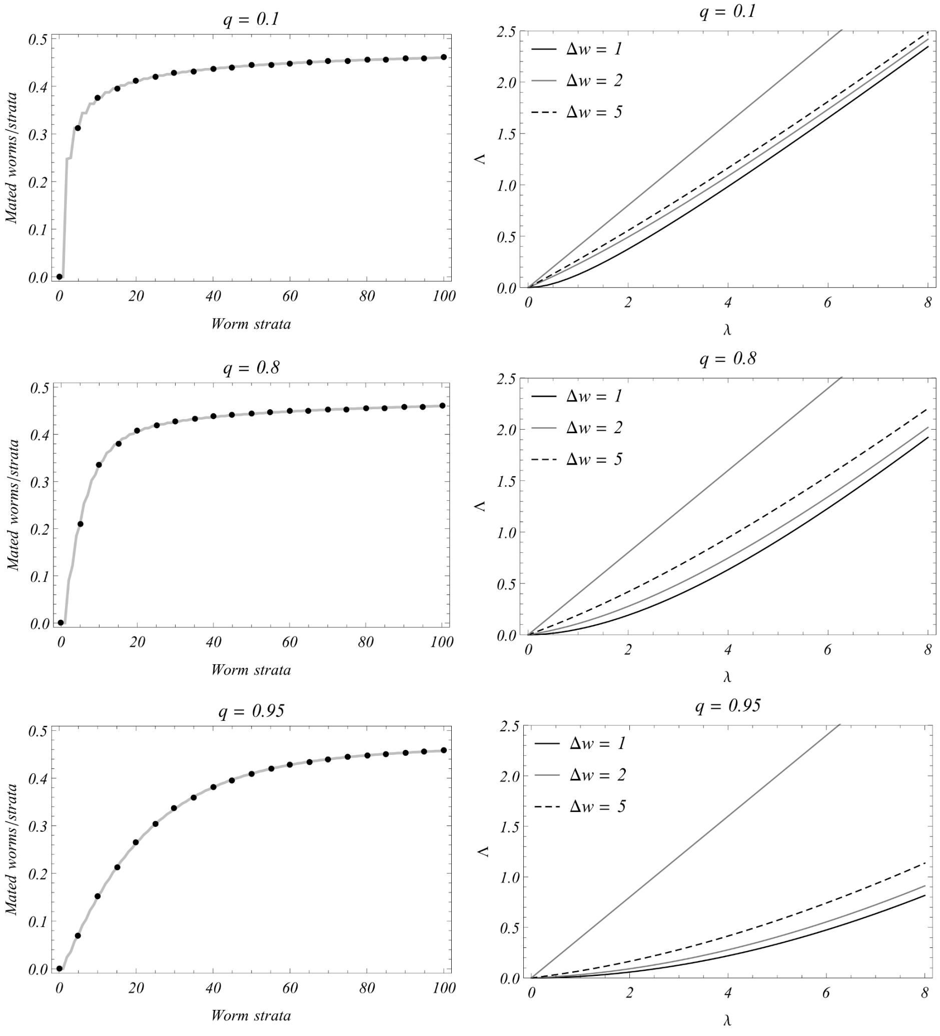

Fig. 5. Effect of SWB inputs (the Δw increment, and the mating hurdle q) on predicted outputs. Left-hand panels show the distribution of mated pairs in host strata, and right-hand panels show the force of snail infection Λ. The three plots in each column correspond to different choices of *q*. Column (a) compares mated fraction ϕ_n of equation (20) for fine-grain system $\Delta w = 1$ (gray line) vs. coarse-grained $\Delta w = 5$ (black dots). Step Δw has only a minor effect on fraction ϕ_n (*i.e.,* low sensitivity), but the force of infection $\Lambda(\lambda, q, \Delta w)$ (column (b)) shows more sensitivity to Δw (higher Δw predicts stronger force, particularly at low λ). Overall, the mating effect on Λ is significant - all curves in (b) depart from the simple linear relation $\Lambda = B\,\lambda$ (the thin, straight line), and increased hurdle factor q also has a significant effect in lowering Λ.

doi:10.1371/journal.pone.0115875.g005

$$\phi_n(\Delta w, q) = \frac{\psi\left(n\,\Delta w, q^{\Delta w}\right)}{n\,\Delta w} \tag{20}$$

with mating factor $\psi(n, q)$ given by (14). As n increases, ϕ_n approaches its maximal value, 1/2, at a q-dependent relaxation rate. Fig. 5′s left hand panels show the differential q effect, whereby an increasing hurdle factor from 0.1 to 0.95

would significantly slow the system's relaxation rate by 1/2. The immediate effect of the reduced mating capacity at low n is a significant reduction of FOI, Λ (right hand panels of Fig. 5). As explained below, this behavior is primarily responsible for the breakpoint phenomena in SWB systems.

Turning to equilibrium analysis of the SWB system (13), under prescribed FOI, population turnover, and population sources, we use rescaled values (λ, μ, S) over worm mortality, γ. As mentioned, no analytic solutions for (13) exist except the limiting case $\mu=0$, $S=0$ (stationary host population without turnover). Here $\{h_n^*(\lambda,0)\}$ becomes the principal (zero) eigenvector of the Frobenius-type matrix A of (13)

$$A=\begin{bmatrix} -\lambda & 1 & 0 & \cdots & 0 \\ \lambda & -\lambda-1 & 2 & 0 & \vdots \\ 0 & \lambda & -\lambda-2 & \ddots & 0 \\ \vdots & \ddots & \ddots & \ddots & n \\ 0 & \cdots & 0 & \lambda & -\lambda-n \end{bmatrix}$$

which gives $h_n^*(\lambda, 0)=e^{-\lambda}\dfrac{\lambda^n}{n!}$ - a Poisson distribution. We expect $\{h_n^*(\lambda, \mu)\}$ for small μ could be approximated by the limiting Poisson $h_n^*(\lambda)$.

Fig. 2 shows numeric simulation of for the young-age group with a stationary uninfected source. Panels (a–b) compare NB/Poisson distributions with fixed mean $w=10$, to SWB-distributions $\{h_n^*(\lambda, \mu)\}$ with fixed $\lambda=10$, across a range of NB aggregation values k. They produce similar patterns, whereby clumping increases as $k\rightarrow\infty$ (for NB), and $\mu\rightarrow 0$ (for SWB). In that sense, the dimensionless SWB-turnover time $1/\mu$ plays the same role as NB-aggregation k. Note that realistic demographic values are relatively small $\mu=.1-.2$ (based on 20–40 year human life span [41], vs. worm $1/\gamma=4$ years [33]). As expected from the $\mu=0$ case, they look like Poisson distribution results with mean $m\approx\lambda$; Fig. 2 (c–d) demonstrate this for $\mu=.1$. We observe closely matched Poisson cases (c) and "small μ" SWB cases (d), whereas large μ cases (b) correspond to increased NB aggregation k. We conclude that in the absence of other confounding factors, a simple (homogeneous) SWB host system with an uninfected population source would attain a Poisson-like equilibrium state with $w\approx\lambda$.

The last panel, 2(f), shows infection zero-prevalence $\Omega(\lambda, \mu)$ for several values, μ. Once again, we note parallels with the corresponding MacDonald NB prevalence functions for increased k (panel 2(e)).

Turning to fully coupled SWB + snail systems, equilibria can be computed from the reduced snail equation, described by function $F_{SW}(y)$

$$F_{SW}(y)=\frac{B}{\nu}\cdot\sum\nolimits_{n\geq 1}\psi\left(n\,\Delta w,\, q^{\Delta w}\right)\cdot h_n{}^*(\lambda,\, \mu)\cdot(1-y)-y \tag{21}$$

which plays a similar role to Macdonald function $F(y,\ldots)$, (equation 19).

In the Macdonald case, $F(y)$ undergoes a transition from "stable zero" to a "bistable" (breakpoint) state, depending on model parameters, and zero is always a stable equilibrium.

Our analysis of SWB (S4 Appendix in S1 Text) reveals a more complicated picture. Specifically, we identified three cases: (i) single stable zero (eradication); (ii) double stable/unstable pair ("zero + endemic"), as in the Macdonald MWB system without mating ($\phi=1$); and (iii) a bistable (zero-breakpoint-endemic) case, like the mated extended-MWB case. The outcome depends on transmission rates (A, B) and the mating hurdle $0\leq q<1$. Unlike the Macdonald case (see Fig. 4(b)), the parameter space is now divided into 3 regions. A condition for a stable zero equilibrium ($y^*=0$) is negative slope $\frac{dF_{SW}}{dy}(0)<0$. The slope can by computed in terms of parameters A,B, q, and the worm-step Δw used in SWB formulation (see S4 Appendix in S1 Text), namely.

$$\frac{d}{dy}F_{SW}(0)=R_0\,\psi_{\Delta w}-1<0 \tag{22}$$

where R_0 is the standard Macdonald BRN (18) adjusted for population turnover ($\gamma\to\gamma+\mu$) and ψ_n- mated fraction (2). In the simulations described below, we used step $\Delta w=2$. Condition (22) is similar to stability of the "zero" equilibrium ($R_0<1$) for a simple Macdonald system ($\phi=1$). In that sense, the SWB system occupies an intermediate place between two Macdonald types: the simple "no mating" ($\phi=1$), and the "breakpoint" ($\phi<1$) containing system.

A more challenging task was to identify stable endemic regions in the (A, B, q)-parameter space of SWB. Unlike Macdonald $F(y)$, the SWB $F_{SW}(y)$ has no simple algebraic form, so we computed it numerically (see S4 Appendix in S1 Text). The results are shown in Fig. 6: the shaded region in the A,B plane marks the bistable (breakpoint) parameter values, above this shaded area, the system is "stable endemic", below the area, it goes to "stable zero" (eradication). We observe that having an increased q would expand the breakpoint region, and shift it up in the (A, B) plane. Qualitatively, the stable endemic regions of the Macdonald system in Fig. 4(b) and those of the SWB (Fig. 6) look similar.

Fig. 7. demonstrates $F_{SW}(y)$ for fixed rates $A=6$, $B=5.8$ and several q (above, in, and below the breakpoint region) to observe all three equilibrium patterns. Their profiles resemble MacDonald-MWB functions $F(y)$ of Fig. 4 (panels 4c and 4d) within the breakpoint parameter ranges (dashed and light gray curves), but they look qualitatively similar to the simple (no-mating) MacDonald function $F(y)$ (black curve for $q=.95$) above the shaded region in Fig. 6(c). Panel 7(b) shows the resulting endemic and breakpoint equilibrium distributions $\{h_n^*\}$ in the breakpoint case (a) $q=.95$, the former being more aggregated with higher MWB value.

Overall, a significant finding of this SWB modeling is that the breakpoint regions occupy a relatively small fraction of (A, B, q) space. So, randomly chosen A, B would most likely result in a simple Macdonald-type dichotomy: either

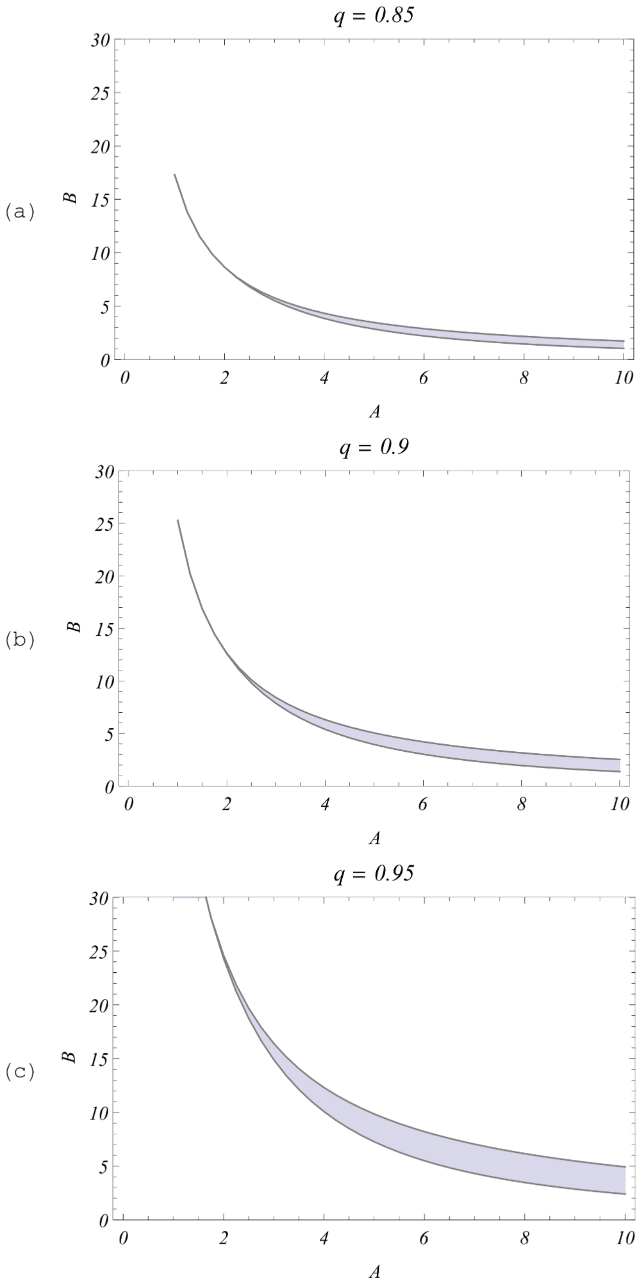

Fig. 6. Stability regions of the coupled SWB system (13)–(16) for the young-age group. Shown are values for ($\mu = .2$) in *A, B* - parameter space at different values of mating hurdle *q*. The shaded region in each plot marks the bistable (zero-breakpoint-endemic) range; all values above it are saddle-nodes (''unstable zero + stable endemic''); below it is the region of ''stable zero'' (eradication).

doi:10.1371/journal.pone.0115875.g006

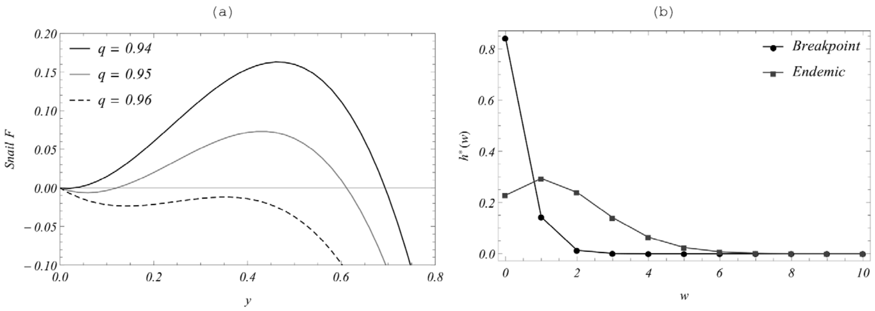

Fig. 7. Equilibrium patterns for function F_{SW}. Panel (a) shows equilibrium snail function, $F_{SW}(y)$, with transmission rates $A=6$, $B=5.8$, exhibiting three equilibrium patterns: saddle-node (zero - endemic), bistable (breakpoint) and stable "zero", for increasing mating hurdle q. Panel (b) shows SWB equilibrium distributions for the stable (endemic) equilibrium and unstable breakpoint for the middle curve $q=.95$ of panel (a).

doi:10.1371/journal.pone.0115875.g007

"stable zero" or "stable endemic". In the real world, however, situations *A, B* might be related, and the breakpoint could actually play a more significant role in determining *Schistosoma* persistence. This prompted us to explore their dynamic implications.

Dynamic responses and projected effects of drug treatment

To reflect the impact of control programs in endemic *Schistosoma* transmission settings, it is important to compare the respective dynamic responses of the MWB and SWB models, including their endemic equilibria, relaxation patterns, and the long term impact of control interventions. While there are some major differences in their structure, the two models have some similarities in terms of a comparable set of parameters: *A*, *B*, aggregation *k*, for MacDonald; and *A*, *B*, and the mating hurdle, *q*, for SWB. One way to compare the two types of model is to calibrate them with an identical data set and conduct numeric simulations of control. (The calibration procedures are outlined in S5 Appendix in S1 Text).

Drug treatment with praziquantel clears a sizable fraction of adult worms (up to 90–95%), and thereby reduces worm burden in treated populations. There are two essential parameters of mass drug administration (MDA): i) drug efficacy $0< \ <1$ - in terms of the fraction of surviving worms $(=.05-.1)$, and ii) the human population fraction covered by treatment $0<f<1$. Other important factors in program efficacy are the frequency (timing) and the number of MDA sessions. Mathematically, MDA is implemented differently in the two different types of model.

Let us note that our data set was chosen in a special way, to produce a breakpoint-type SWB system. Fig. 8 shows two reduced equilibrium functions: MacDonald's $F(y)$ of equation (19) (shown in gray), and the SWB-function $F_{SW}(y)$ of equation (21) (in black). Both exhibit breakpoints near $y=0$, but SWB has higher value y_B^* than MacDonald's (see Table 5). It suggests that SWB infection would be easier (faster) brought to elimination compared to MacDonald case.

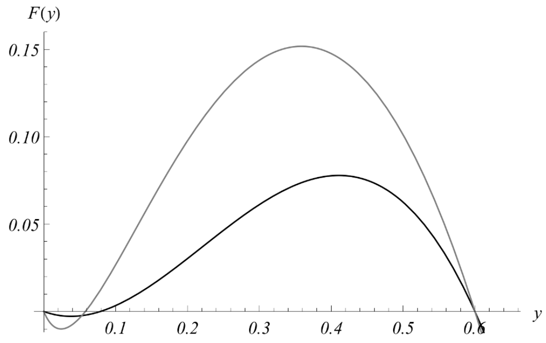

Fig. 8. Equilibrium functions for the two types of model. Shown are $F(y)$: MacDonald equation (19) (gray), and SWB equation (21) (black), for calibrated model parameters of Table 4

doi:10.1371/journal.pone.0115875.g008

MDA for Macdonald-type systems

For the Macdonald-MWB system, one divides population into treated+ untreated groups, with burden $v(t)$ - treated, $u(t)$ - untreated) and sets up an extended version of system (9) for variables $\{u,v,y\}$

$$\begin{aligned}\frac{du}{dt} &= A\cdot y - \gamma\cdot u \\ \frac{dv}{dt} &= A\cdot y - [\gamma + (1-)\delta(t-t_0)]\cdot v \\ \frac{dy}{dt} &= \Lambda(u,\, v)\cdot(1-y) - v\cdot y\end{aligned} \tag{23}$$

Here worm mortality for the treated group undergoes an abrupt change at the treatment time t_0, $\gamma \to \gamma + (1-)\delta(t-t_0)$, represented by Dirac delta-function $\delta(t-t_0)$. The combined force of snail infection by the two groups is given by

$$\Lambda(u,\, v) = B\cdot\left[(1-f)\cdot\phi(u,f)\frac{u}{2} + f\cdot\phi(v,\, k)\frac{v}{2}\right]$$

Table 5. Calibrated model parameters from the data inputs listed in Table 4.

	$a=A/\gamma$	$b=B/v$	***k***	***q***	w^*	λ^*	**Breakpoint** y_B^*
Extended MacDonald MWB model employing NB distribution	2.3	3.27	.14		1.37		.057
Simple MacDonald MWB model	1.5	3.27					
SWB model with mating	6	3.27		.9		1.8	.08
Simple SWB model	1.7	3.27				.51	

(*abbreviations:* MWB, mean worm burden; NB, negative binomial;, SWB, stratified worm burden. Details of calibration approach are given in S5 Appendix in S1 Text. SWB systems have increment $\Delta w=2$. For MacDonald systems, $a=\frac{A}{\gamma+\mu}$.

doi:10.1371/journal.pone.0115875.t005

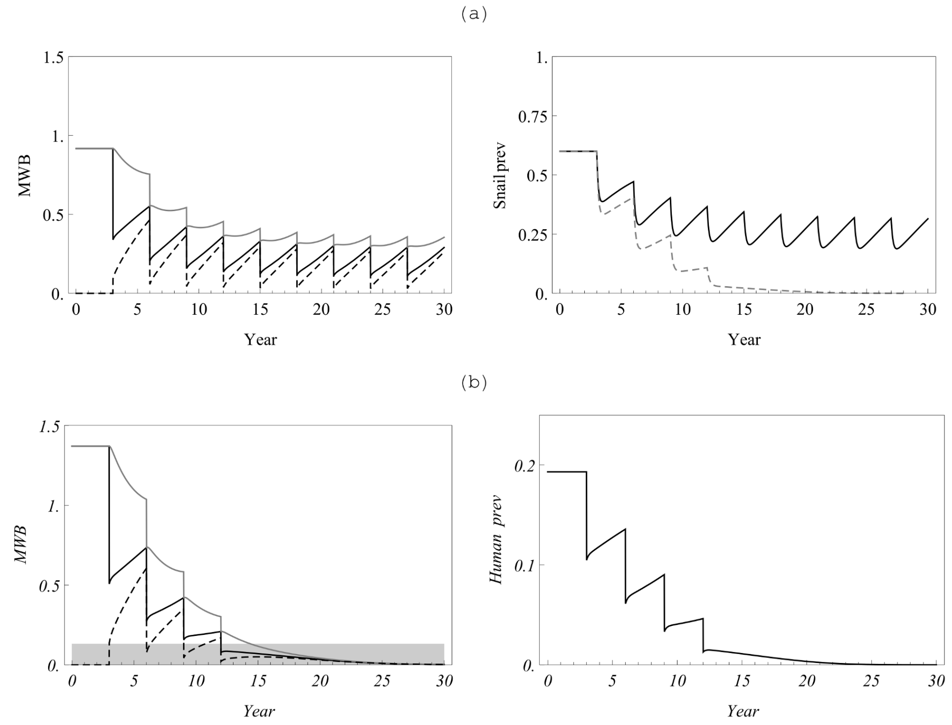

Fig. 9. A multi-year treatment cycle with 70% population coverage and drug efficacy of 90%. Panels indicate results for (a) a simple Macdonald MWB model without mating; (b) a MWB model with NB distribution and an included mating function (equation (36), S3 Appendix in S1 Text) having breakpoint level $w_0 = .13$ (shaded range, lower left hand panel). On both left hand plots, solid black is the overall community MWB, $w(t) = (1-f)u(t) + f\,v(t)$; gray is the untreated group $u(t)$, and the dashed line is the treated group $v(t)$. The upper right hand panel indicates yearly snail prevalence of infection without (solid line) or with (dashed line) inclusion of the mating factor, and hence the breakpoint, in the model. The lower right hand panel indicates expected human prevalence with MDA treatment in the breakpoint setting.

doi:10.1371/journal.pone.0115875.g009

Another way to implement it numerically is to terminate solution (23) at $t = t_0$, and reinitialize using MWB variable $w(t) = (1-f)\cdot u(t) + f\cdot v(t)$, as

$$\begin{aligned} u|_{t_0} &= w(t_0) \\ v|_{t_0} &= \cdot w(t_0) \end{aligned} \tag{24}$$

In practice, this means that population is randomly divided into treated/untreated fractions in each session and no prior treatment data are incorporated. Such schemes can be repeated any number of times ($t_0 < t_1 < \ldots$) with prescribed frequency, and prescribed treatment fractions (f_0; f_1; ...). Furthermore, these schemes could be augmented with additional features of monitoring and control, *e.g.*, control termination after infection levels are brought below a specified level (the natural choice would be a 'breakpoint').

Fig. 9 compares a hypothetical MDA control program, having 70% coverage and a 90% cure rate, for two calibrated MacDonald systems: simple $\phi(w) = 1$

(panel (a)) *vs.* a modified NB-MacDonald with mating function $\phi(w, k)$(panel (b), see equation (36) in S3 Appendix in S1 Text). Both systems are initialized at their endemic states. The former scenario indicates that the region would require an indefinite series of treatments to maintain control, while the latter suggests that MDA would bring the system to eradication after 4 sessions, when $w < .13$, drops below the breakpoint value.

SWB drug control

The effect of drug treatment on the SWB model is to move a treated fraction from higher strata $h_n(t)$ to lower-level strata $h_m(t)$, with $m \approx n$ determined by the drug efficacy [23]. In particular, all strata having $\{h_m : 0 \le m < 1/\}$ would shift to h_0 (complete clearing), the next higher range $\{h_m : 1/ \le m < 2/\}$ would go to h_1 etc. The corresponding MDA reinitialization event then becomes

$$h_0|_{t_0} = (1-f)h_0 + f \sum_{0 \le m < 1/} h_m(t_0)$$

$$h_1|_{t_0} = (1-f)h_1 + f \sum_{1/ \le m < 2/} h_m(t_0)$$

$$h_1|_{t_0} = \ldots$$

However, the snail equation (force of infection Λ) doesn't change its functional form as variables $\{h_n(t)\}$ get reshuffled.

To examine the effect of control on long term SWB histories we took the calibrated SWB system with parameters of Table 4 and ran the same four-year control strategy as for MWB described above. The results of simulation are compared to the calibrated MacDonald-MWB system in Fig. 10(a). Overall, SWB simulations predict more efficient reduction of worm burden and prevalence in the four-session control program. Both systems predict elimination below their breakpoints.

The concerning feature of SWB prediction is its high sensitivity to the Allee parameter q. A slight change from $q = .9$ (breakpoint case I) to $q = .87$ (saddle-node case II), has important long-term implications, shown in Fig. 10(b). In both case I and case II, MWB can be brought to relatively low levels after 4 sessions, but case I goes to extinction while case II relaxes back to pretreatment endemic levels – *i.e.*, a finite eradication time for case I vs. the requirement for indefinitely sustained effort for case II.

Conclusions

Uneven parasite burden and sex distribution have a significant impact on infection levels and sustainability of transmission. Indeed, both depend on

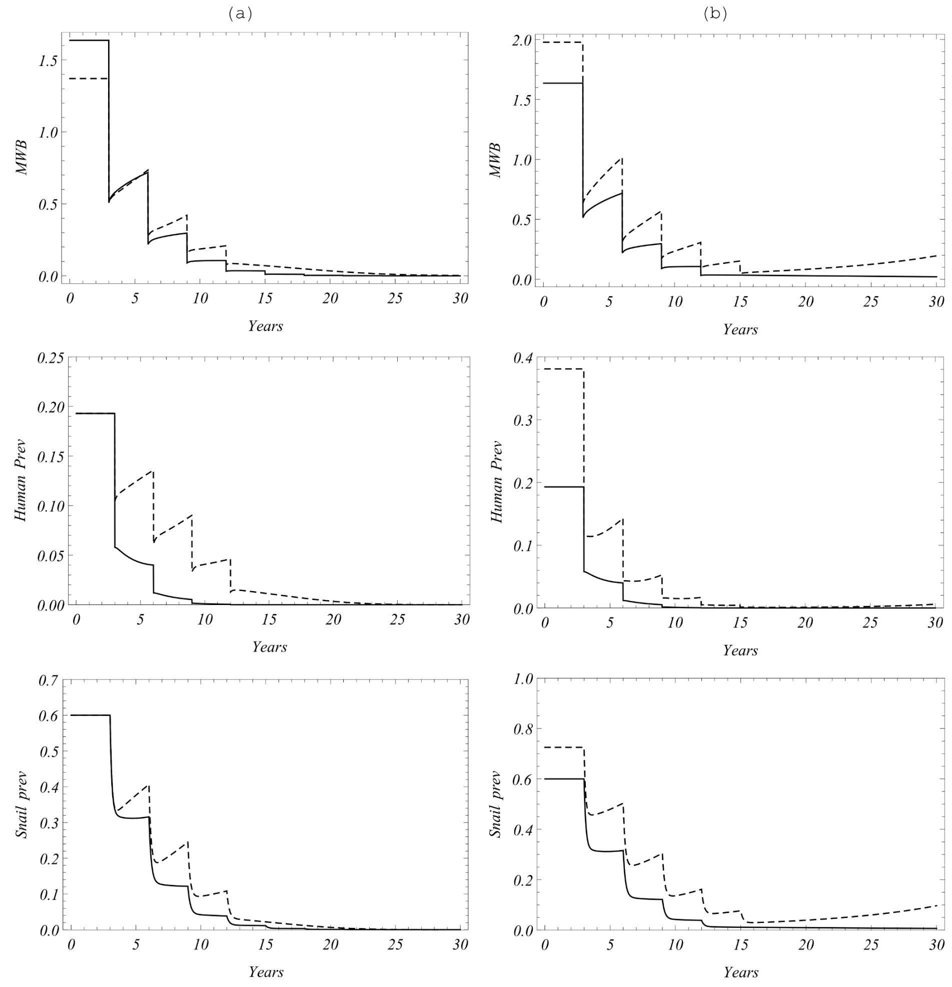

Fig. 10. Control of the calibrated SWB system with the same MDA strategy as for previous figure. The left column (a) compares SWB outputs (mean worm load, human and snail prevalence) in the solid curves, compared with the MWB model outputs shown in the dashed curves. SWB predicts faster (more efficient) reduction of all infection outcomes. Panel (b) compares the SWB system of column (a) (solid) with an MDA-perturbed system where mating hurdle was changed to $q = .87$ (dashed). The perturbed system has no breakpoint (which has sensitive dependence on q in the SWB system) and after an initial four-treatment reduction, infection gradually relaxes to the pre-MDA endemic state

doi:10.1371/journal.pone.0115875.g010

fertilized female count, and uneven parasite loads create hurdles for worm mating that reduce resulting egg production, particularly in low-level worm burden host strata. May and colleagues [12, 18] have addressed these issues in the context of Macdonald MWB formulation, utilizing an assumption about infection distribution patterns (*e.g.*, NB, Poisson) to facilitate solutions to their model. This analysis produced a modified Macdonald-MWB system that includes a "mating factor". The mating factor makes profound changes in mathematical structure and equilibria of Macdonald-type system, creating a breakpoint between its "zero" and endemic levels. Hence, a modified Macdonald system (with mating),

unlike its simple cousin, predicts elimination after a finite number of control interventions.

Consideration of the MWB work raises several questions: how reasonable is the NB assumption?; should NB aggregation parameters be fixed or subject to change?; how can these models be reconciled with underlying host demographics?; and how reliable are assumptions about the effects of interventions (MDA) on model dynamics and parameters? In place of the MWB approach, we believe that a proper way to account for mating and uneven distribution is through a stratified worm burden approach (SWB) to model development. Mathematically, worm strata can be viewed as population level distribution function (PDF) of the underlying stochastic process of worm acquisition/loss.

Some important conclusions of the MWB-SWB comparative analysis:

i) In the modified MacDonald-MWB systems, the NB aggregation parameter k should not be treated as fixed, but should be treated as a dynamic variable. When such systems are calibrated based on equilibrium relations, the outcomes are highly sensitive to k. Furthermore, k-values can undergo significant changes after intervention.

ii) A consistent dynamic formulation (based on the underlying SWB) requires an extension of the Macdonald system with either k as an additional variable, or an equivalent ''second-moment'' variable (where the MWB *w* represents its ''first moment'').

iii) However, this extended (1st+2nd moment) system has inherent inconsistencies when coupled to host demographics. The consistent formulation is possible only within a limited context - when the sole source of host population enters the uninfected (''zero-level'') SWB strata, *e.g.*, as newborn children. So one can provide an extended Macdonald system for a children's age category (with a newborn source), but not for a corresponding adult group or for any coupled ''child-adult'' systems. By contrast, the SWB system is free of such limitations and can be set for any age- or location-structured populations.

iv) In exploring the breakpoint phenomena for both MWB (fixed k) and SWB systems, in the former case (MWB) a breakpoint comes automatically in any setting with a positive endemic level, *i.e.*, with sufficiently high transmission rates (*a, b*); in the latter case (SWB), zero and endemic equilibria are not rigidly connected, so there are three possible outcomes: ''zero-breakpoint-endemic'', ''zero-endemic'', and ''zero''. The existence of breakpoint depends on an additional (low-density) mating constraint. Here we have accounted for such an effect by a single parameter q – the probability of mating failure per single adult. We showed that in the SWB system, the breakpoint phenomena depends strongly on q and we have explored the bounds of breakpoint regions in the (*a, b, q*)-parameter space.

v) In both cases, (MacDonald and SWB), we can explore the effect of drug treatment (MDA) with different parameter values (coverage fraction, drug efficacy, treatment frequency/year). In all cases we tested, a breakpoint was

shown to bring elimination after a finite number of interventions whenever infection levels fell below the breakpoint value.

Our study of mating, breakpoints, and infection persistence raises several issues on the role and meaning of BRN in transmission models. BRN by itself can predict a transition from "no infection" to "endemic state" (above/below critical value), but has no direct links to "breakpoints". Mathematically the "breakpoint phenomena" amounts to a two-parameter space analysis. It has clear implications for parasite elimination: MDA, by itself, won't necessarily affect BRN (transmission environment), but by driving the system below an inherent breakpoint, we might achieve the requisite transition (bifurcation) to zero endemicity. Future work with extended control program datasets and improved SWB methodology will shed the light on the probable existence of breakpoints for elimination. In extending our published work [23], we plan to apply the newly extended SWB methodology to model structured host populations and distributed human -snail environments [19, 20]. We believe that the modified SWB approach will provide more accurate and reliable prediction than the conventional MacDonald MWB-based methods.

Supporting Information

S1 Text. S1–S5 Appendices. Extended description and formulae detailing: S1 Appendix) The background on the problems of *Schistosoma* transmission model formulation; S2 Appendix) Moment equations for SWB, and the extended MWB model; S3 Appendix) Mating function in Macdonald and SWB systems; S4 Appendix) Equilibria of a coupled human SWB - snail system; and S5 Appendix) Model calibration.
doi:10.1371/journal.pone.0115875.s001 (DOCX)

S1 Workbook. Mathematica software notebook file (.nb) with programming for MWB and SWB transmission models.
doi:10.1371/journal.pone.0115875.s002 (NB)

Author Contributions

Conceived and designed the experiments: DG CHK. Performed the experiments: DG CHK. Analyzed the data: DG CHK. Contributed reagents/materials/analysis tools: DG CHK. Contributed to the writing of the manuscript: DG CHK.

References

1. **King CH, Sturrock RF, Kariuki HC, Hamburger J** (2006) Transmission control for schistosomiasis - why it matters now. Trends Parasitol 22: 575–582.

2. **Colley DG, Bustinduy AL, Secor WE, King CH** (2014) Human schistosomiasis. Lancet 383: 2253–2264.

3. **Bustinduy AL, King CH** (2009) Parasitic Helminths. In: Fratamico PM, Smith JL, Brogden KA, editors. Post-Infectious Sequelae and Long-Term Consequences of Infectious Diseases. Washington: American Society for Microbiology Press. 291–329.

4. **Warren KS** (1982) Selective primary health care: strategies for control of disease in the developing world. I. Schistosomiasis. Rev Infect Dis 4: 715–726.

5. **Jordan P, Christie JD, Unrau GO** (1980) Schistosomiasis transmission with particular reference to possible ecological and biological methods of control. A review. Acta Trop 37: 95–135.

6. **Savioli L, Daumiere D** (2012) Accelerating Work to Overcome the Global Impact of Neglected Tropical Diseases: A Roadmap for Implementation. Geneva: World Health Organization.

7. **MacDonald G** (1965) The dynamics of helminth infections, with special reference to schistosomes. Trans R Soc Trop Med Hyg 59: 489–506.

8. **Coura-Filho P, Mendes NM, de Souza CP, Pereira JP** (1992) The prolonged use of niclosamide as a molluscicide for the control of *Schistosoma mansoni*. Rev Inst Med Trop Sao Paulo 34: 427–431.

9. **Duke BO, Moore PJ** (1976) The use of a molluscicide, in conjunction with chemotherapy, to control *Schistosoma haematobium* at the Barombi Lake foci in Cameroon. I. The attack on the snail hosts, using N-tritylmorpholine, and the effect on transmission from snail to man. Tropenmed Parasitol 27: 297–313.

10. **Fenwick A** (1970) The development of snail control methods on an irrigated sugar-cane estate in northern Tanzania. Bull World Health Organ 42: 589–596.

11. **Nåsell I** (1978) Mating models for schistosomes. Journal of Mathematical Biology 6: 21–35.

12. **May RM** (1977) Togetherness among schistosomes: its effects on the dynamics of the infection. Mathematical Biosciences 35: 301–343.

13. **Coura Filho P, Rocha RS, de Lima ECMF, Katz N** (1992) A municipal level approach to the management of schistosomiasis control in Peri-Peri, MG, Brazil. Rev Inst Med Trop Sao Paulo 34: 543–548.

14. **Fenwick A, Jorgensen TA** (1972) The effect of a control programme against *Schistosoma mansoni* on the prevalence and intensity of infection on an irrigated sugar estate in northern Tanzania. Bull World Health Organ 47: 579–586.

15. **Barnish G, Jordan P, Bartholomew RK, Grist E** (1982) Routine focal mollusciciding after chemotherapy to control *Schistosoma mansoni* in Cul de Sac valley, Saint Lucia. Trans R Soc Trop Med Hyg 76: 602–609.

16. **Clarke VV, Blair DM, Weber MC** (1973) The Mayfield experiment: an attempt to eliminate bilharziasis from a small community by repeated treatment of the infected people combined with intensive snail control. Cent Afr J Med: 14–22.

17. **Nåsell I, Hirsch WM** (1973) The transmission dynamics of schistosomiasis. Communications on Pure and Applied Mathematics 26: 395–453.

18. **Bradley DJ, May RM** (1978) Consequences of helminth aggregation for the dynamics of schistosomiasis. Trans R Soc Trop Med Hyg 72: 262–273.

19. **Woolhouse ME, Chandiwana SK** (1989) Spatial and temporal heterogeneity in the population dynamics of *Bulinus globosus* and *Biomphalaria pfeifferi* and in the epidemiology of their infection with schistosomes. Parasitology 98: 21–34.

20. **Woolhouse ME, Etard JF, Dietz K, Ndhlovu PD, Chandiwana SK** (1998) Heterogeneities in schistosome transmission dynamics and control. Parasitology 117: 475–482.

21. **May RM, Woolhouse ME** (1993) Biased sex ratios and parasite mating probabilities. Parasitology 107: 287–295.

22. **Gurarie D, King CH** (2005) Heterogeneous model of schistosomiasis transmission and long-term control: the combined influence of spatial variation and age-dependent factors on optimal allocation of drug therapy. Parasitology 130: 49–65.

23. **Gurarie D, King CH, Wang X** (2010) A new approach to modelling schistosomiasis transmission based on stratified worm burden. Parasitology 137: 1951–1965.

24. **Barbour AD** (1996) Modeling the transmission of schistosomiasis: an introductory view. Am J Trop Med Hyg 55: 135–143.

25. **Thomas JD** (1973) Schistosomiasis and control of molluscan hosts of human schistosomes with particular reference to possible self-regulatory mechanisms. Adv Parasitol 11: 307–394.

26. **Ribeiro-Paes JT, Rodrigues V** (1997) Sex determination and female reproductive development in the genus *Schistosoma*: a review. Rev Inst Med Trop Sao Paulo 39: 337–344.

27. **Dobson A, Roberts M** (1994) The population dynamics of parasitic helminth communities. Parasitology 109 Suppl: S97–108.

28. **Shaw DJ, Grenfell BT, Dobson AP** (1998) Patterns of macroparasite aggregation in wildlife host populations. Parasitology 117 (Pt 6): 597–610.

29. **Morand S, Pointier J-P, Borel G, Théron A** (1993) Pairing probability of schistosomes related to their distribution among the host population. Ecology 74: 2444–2449.

30. **Anderson RM, May RM** (1978) Regulation and stability of host-parasite population interactions. I. Journal of Animal Ecology 47: 219–247.

31. **Anderson RM, May RM** (1978) Regulation and stability of host-parasite population interactions. II. Journal of Animal Ecology 47: 249–267.

32. **Wang X, Gurarie D, Mungai PL, Muchiri EM, Kitron U, et al.** (2012) Projecting the long-term impact of school-or community-based mass-treatment interventions for control of *Schistosoma* infection. PLoS Neglected Tropical Diseases 6: e1903.

33. **Anderson RM, May RM** (1991) Infectious Diseases of Humans. Oxford: Oxford University Press. 757 p.

34. **Sturrock RF** (1973) Field studies on the transmission of *Schistosoma mansoni* and on the bionomics of its intermediate host, *Biomphalaria glabrata*, on St. Lucia, West Indies. Int J Parasitol 3: 175–194.

35. **Chan MS, Guyatt HL, Bundy DA, Booth M, Fulford AJ, et al.** (1995) The development of an age structured model for schistosomiasis transmission dynamics and control and its validation for *Schistosoma mansoni*. Epidemiol Infect 115: 325–344.

36. **Chan MS, Guyatt HL, Bundy DAP, Medley GF** (1994) The development and validation of an age-structured model for the evaluation of disease control strategies for intestinal helminths. Parasitology 109: 389–396.

37. **Cornell SJ** (2010) Modelling stochastic transmission processes in helminth infections. In: Michael E, Spear RC, , editors. Modelling Parasite Transmission and Control: Springer. 66–78.

38. **Pugliese A** (2000) Coexistence of macroparasites without direct interactions. Theor Popul Biol 57: 145–165.

39. **Prichard RK, Basáñez MG, Boatin BA, McCarthy JS, García HH, et al.** (2012) A research agenda for helminth diseases of humans: intervention for control and elimination. PLoS Neglected Tropical Diseases 6: e1549.

40. **Churcher TS, Ferguson NM, Basáñez MG** (2005) Density dependence and overdispersion in the transmission of helminth parasites. Parasitology 131: 121–132.

41. **Warren KS, Mahmoud AAF, Cummings P, Murphy DJ, Houser HB** (1974) Schistosomiasis mansoni in Yemeni in California: duration of infection, presence of disease, therapeutic management. Am J Trop Med Hyg 23: 902–909.
